# Combination of YAP inhibition and photodynamic therapy induces dual DNA damage and activates STING pathway to enhance immunotherapy in uveal melanoma

**DOI:** 10.1016/j.redox.2025.103965

**Published:** 2025-12-06

**Authors:** Shuyang Zhang, Meijiao Song, Jialu Zhang, Jianshu Bai, Xinyu Cao, Lei Zhu, Rui Tian

**Affiliations:** a Department of Ophthalmology, the Second Hospital of Jilin University, Changchun, Jilin Province, 130000, China; b Winship Cancer Institute, Department of Surgery, Emory University School of Medicine, Atlanta, GA, 30322, United States

**Keywords:** Uveal melanoma, Immunotherapy, DNA damage, STING, Verteporfin, HANP

## Abstract

Uveal melanoma (UM) is the most common primary intraocular malignancy in adults and is highly aggressive, with no therapies shown to improve overall survival. Although immunotherapy achieves response rates of 33–40 % in advanced cutaneous melanoma, its efficacy in UM is disappointingly low, with only 3.6 % of patients responding. Herein, we report a nanomedicine-based strategy to enhance immunotherapy efficacy in UM by strong activation of the cyclic guanosine 3′,5′-cyclic monophosphate–adenosine monophosphate synthase (cGAS)–stimulator of interferon genes (STING) pathway through dual deoxyribonucleic acid (DNA) damage induced by Yes-associated protein (YAP) inhibition and photodynamic therapy (PDT). This approach, using hyaluronic acid nanoparticle (HANP)-formulated verteporfin (HANP/VP), concurrently induced nuclear- and mitochondrial-DNA damage, promoted immunogenic cell death (ICD), and drove T-lymphocyte infiltration into the tumor microenvironment (TME). When combined with anti–programmed death-ligand 1 (anti–PD-L1) antibody (Ab) and laser radiation (690 nm, 200 mW/cm^2^, 10 min), HANP/VP significantly increased production of the pro-inflammatory cytokines interferon-γ (IFN-γ), IFN-β1, and tumor necrosis factor-α (TNF-α), enhanced dendritic-cell (DC) maturation and Cluster of Differentiation 8–positive (CD8^+^) T-cell expansion, suppressed tumor growth by 96.20 ± 7.22 %, and extended survival from 33 to >80 days in orthotopic UM models. Importantly, rechallenge experiments confirmed durable antitumor immunity and prevention of UM recurrence. Overall, our findings established HANP/VP as a multifunctional nanomedicine that reprogrammed the TME and elicited potent antitumor immunity through dual DNA damage and STING activation. The study highlights a promising translational strategy for overcoming immunotherapeutic resistance in UM and converting immunologically “cold” tumors into “hot” ones, thereby improving responses to immune checkpoint blockade (ICB).

## 1 Introduction

Uveal melanoma (UM) is the most common primary intraocular malignancy in adults and demonstrates highly aggressive biological characteristics [1]. The standard treatment for medium-sized and localized UM is radiotherapy, while advanced cases often require surgery [2]. However, these approaches are highly invasive and can lead to significant vision loss. For small UM tumors (tumor height <4 mm), photodynamic therapy (PDT) is a viable option, offering high efficacy while preserving vision [[3], [4], [5]]. Nevertheless, all current treatments have not improved overall survival, and approximately 50 % of UM patients develop metastatic disease [6], with a median survival time of <12 months [[7], [8], [9], [10], [11]]. Since current systemic therapies offer limited efficacy in managing advanced UM [12], innovative therapeutic strategies that inhibit primary UM tumor growth and control the development of metastasis are urgently needed.

Immune checkpoint blockade (ICB) therapy has shown significant efficacy in several solid tumors such as cutaneous melanoma, heightening anticipation for its use in UM. However, the response rate of single-agent ICB therapy in UM is <10 % [13]. Even dual-ICB therapy results in objective response in only 11.5 % of UM patients, accompanied by a treatment-related adverse-event rate of up to 94.2 % [14], indicating the significant challenges associated with implementing ICB in UM. The poor response of UM to immunotherapy can be attributed to its local immunological characteristics: (i) UM capitalizes on the immune-privileged nature of the eye by sequestering antigens to diminish immune recognition and by inhibiting activation of both innate and adaptive immune responses, thereby evading immune surveillance [15]. (ii) The low tumor mutational burden results in insufficient neoantigen presentation and compromised immune recognition [16]. (iii) The tumor microenvironment (TME) is highly immunosuppressive, which collectively hinder dendritic-cell (DC) maturation and limit local T-cell activation [15,[17], [18], [19]]. These elements establish UM as a “cold” tumor, leading to inadequate antitumor immune response. As novel therapeutic combinations have gained increasing recognition for their potential to overcome treatment resistance, multiple studies have investigated pairing ICB with conventional treatment regimens [[20], [21], [22], [23]]. Unfortunately, combination approaches studied to date have not overcome either intrinsic- or acquired-resistance mechanisms, and disease progression has occurred in all patients. This highlights the urgent need for innovative therapeutic strategies that can reshape the TME and enhance UM's response to immunotherapy.

Innate immunity plays a crucial role in tumor progression and therapeutic response. Specifically, the cyclic guanosine 3′,5′-cyclic monophosphate–adenosine monophosphate (GMP-AMP) synthase (cGAS)–stimulator of interferon genes (STING) pathway is a crucial regulator of antitumor immunity and TME remodeling within innate immune pathways [24]. On detecting anomalous cytosolic DNA, cGAS catalyzes the synthesis of cyclic GMP-AMP, which initiates a phosphorylation cascade involving STING, TRIF-activated TANK binding kinase 1 (TBK1), and interferon regulatory factor 3 (IRF3). This mechanism stimulates synthesis of type I interferons and other cytokines, thereby advancing DC maturation and antigen presentation, augmenting T-cell activation and proliferation, and enabling infiltration of immune cells into tumor tissues [25,26]. Unfortunately, clinical trials of STING agonists either as monotherapies or in combination with ICBs have shown limited success to date, largely due to challenges in delivery, pharmacokinetics, and systemic toxicity [27,28]. As an alternative, considerable effort has focused on activating the cGAS–STING pathway indirectly by inducing DNA damage within tumors [29]. For example, a mitochondria-targeting gold–silica hybrid nanocomplex loaded with a boron dipyrromethene (BODIPY)–derived type I photosensitizer (BDP) was recently reported to induce mitochondrial stress, trigger release of mitochondrial DNA (mtDNA), and activate the cGAS–STING pathway, thereby enhancing antitumor immunity and achieving sevenfold inhibition of tumor growth in a subcutaneous breast cancer model involving PDT [30]. To further strengthen DNA damage and activate STING more strongly, researchers developed a biodegradable near-infrared radiation window II (NIR-II) fluorescent pseudosemiconducting polymer, named TPA-BD. This material alleviated hypoxia and boosted tumor immunity by inducing robust immunogenic cell death (ICD) via PDT, which promoted T-lymphocyte infiltration and converted tumors from “cold” to “hot.” In a subcutaneous-UM mouse model, TPA-BD combined with an anti–programmed death-ligand 1 (anti–PD-L1) antibody (Ab) markedly suppressed tumor growth [31]. Although these approaches have demonstrated promising antitumor efficacy by activating STING, most strategies still rely predominantly on single-route damage to DNA, either nuclear or mitochondrial. Recent efforts have sought to integrate multiple therapeutic modalities, such as chemotherapy and PDT, to induce both nuclear-DNA (nDNA) and mtDNA damage for enhanced STING activation [32], but these strategies often require multiple-drug compositions or multistep-conjugation processes, which complicate scalable manufacturing, compromise batch-to-batch consistency, and ultimately limit their translational potential. Furthermore, the majority of recent studies have been conducted in subcutaneous tumor models, which inadequately recapitulate the complexity of tumor development and fail to capture the unique anatomical and biological features of tumors such as UM.

In our previous work, we successfully constructed hyaluronic acid nanoparticles (HANPs) carrying verteporfin (VP), a photosensitizer, enabling efficient delivery of VP into tumors and effective inhibition of tumor growth under laser in a subcutaneous-UM model [33]. Compared with free VP, HANP/VP exhibited improved biocompatibility, enhanced tumor targeting, and superior antitumor efficacy. These results highlight the importance of elucidating the mechanisms underlying HANP/VP therapy in orthotopic UM tumor, particularly whether HANP/VP–mediated PDT is safe to the retinal structure and whether its therapeutic benefit is from DNA damage–induced tumor cell death and immune activation. Such insights are critical to determining whether HANP/VP can overcome the intrinsic resistance of UM to immunotherapy for future translational studies. In addition to being a photosensitizer, VP is a small-molecule inhibitor of Yes-associated protein (YAP)–transcriptional enhanced associate domain (TEAD) activity that selectively binds YAP, disrupts YAP–TEAD complexes, and downregulates YAP expression [[34], [35], [36], [37]]. Because YAP is essential to preserving nuclear-envelope stability and its inhibition activates the cGAS–STING pathway [38], VP could be used to activate STING without laser. Given that PDT leads to both nDNA and mtDNA damage [30,[39], [40], [41]], we hypothesized that by inducing a “dual DNA damage” effect, HANP/VP could activate the cGAS–STING pathway wherein nDNA damage resulted from YAP inhibition and mtDNA damage from PDT, resulting in fast cytosolic-DNA buildup that could significantly activate the cGAS–STING pathway. Moreover, HANP/VP–mediated PDT also triggers ICD, marked by the production of damage-associated molecular patterns (DAMPs) including high-mobility group box 1 protein (HMGB1), heat shock protein 70 (HSP70), adenosine triphosphate (ATP), and calreticulin (CRT) [[42], [43], [44], [45], [46]]. These DAMPs serve as immunostimulatory signals that facilitate the maturation of DCs and augment adaptive immunological activation to further boost the efficacy of immunotherapy across diverse malignancies [[47], [48], [49], [50]].

**Scheme 1:**
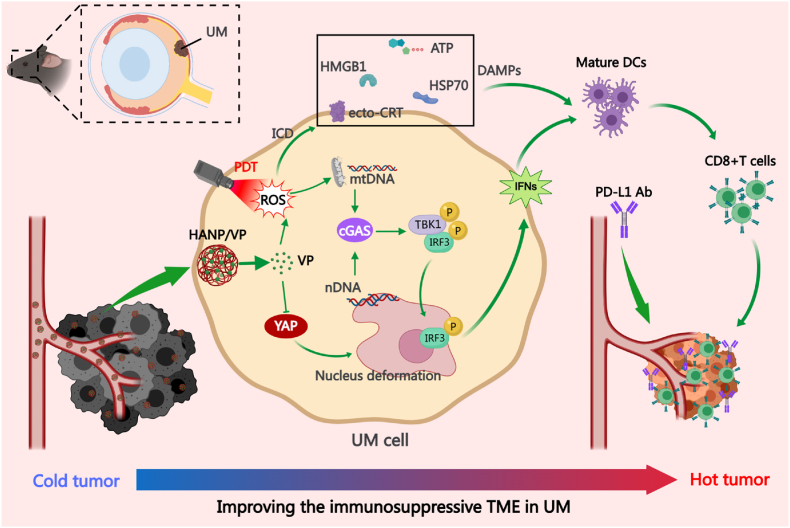
Schematic illustration of the mechanism by which HANP/VP reprograms the immunosuppressive TME in UM through dual DNA damage induced cGAS–STING activation and ICD, thereby stimulating both innate and adaptive immune responses.

In this study, we aimed to elucidate the mechanism by which HANP/VP enhanced antitumor immunity through dual DNA damage and ICD induction, thereby providing a strong mechanistic foundation for overcoming the limited efficacy of immunotherapy in UM (Scheme 1). Specifically, we investigated the effects of HANP/VP on YAP expression, nDNA and mtDNA damage, and subsequent activation of the cGAS–STING pathway and ICD, with or without laser. Furthermore, therapeutic efficacy was evaluated in orthotopic UM models by assessing tumor response and survival following combined treatment with HANP/VP, PD-L1 Ab, and PDT. This study will position HANP/VP as a multifunctional nanocomplex that promotes DC maturation, facilitates immune cell infiltration, and synergizes with PD-L1 blockade. In clinical scenarios where PDT is not feasible, such as UM liver metastases, the combination of HANP/VP-mediated YAP inhibition with ICB can still elicit a strong antitumor immune response, offering a promising therapeutic strategy for metastatic disease. Our ultimate objective is to develop a highly translatable YAP inhibition and PDT functional NP strategy that, by delivering a single small molecule, induces dual DNA damage and ICD to activate STING signaling, boost immunotherapy efficacy, and extend survival in UM.

## 2 Results and discussion

### 2.1 HANP/VP induced mtDNA and nDNA damage via YAP inhibition and PDT

Despite its clinical approval, the liposomal formulation of VP (Visudyne) [51] relies primarily on the enhanced permeability and retention (EPR) effect for tumor accumulation, which frequently leads to heterogeneous and suboptimal delivery in solid tumors [52]. Incorporating active tumor-targeting strategies could substantially improve tumor selectivity and therapeutic efficacy, highlighting the limitations of liposomal VP as an optimal clinical candidate for UM. Hyaluronic acid (HA) has a longstanding history of clinical use in ophthalmology and has been approved by the Food and Drug Administration (FDA) as a component in several ocular formulations [53]. HA is a major component of the vitreous humor and extracellular matrix (ECM) in the eye, and its structural similarity to other native ocular components enables low immunogenicity and enhanced tissue penetration in therapeutic use [54,55]. In addition, HA specifically binds to Cluster of Differentiation 44 (CD44) molecules, which are abundantly expressed on the surfaces of tumor cells [[55], [56], [57]]. Therefore, the HA-based NP has the advantages of both passive EPR effect and active CD44 ability to target tumors.

**Fig. 1:**
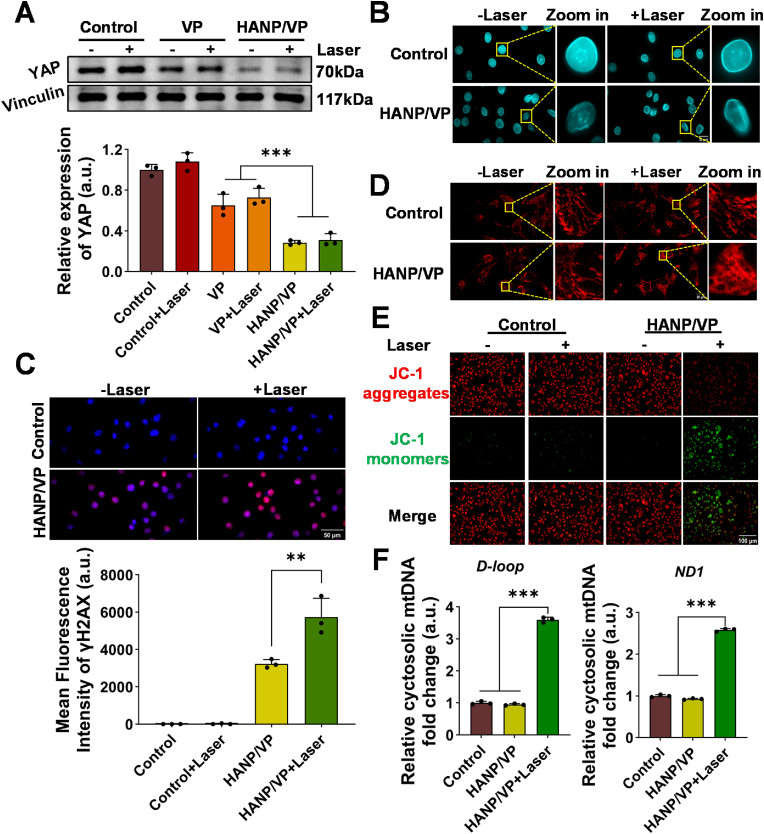
**HANP/VP induces dual DNA damage in vitro via YAP-inhibition and PDT. (A)** The expression level of YAP was detected by Western blot, using Vinculin as an internal reference protein after corresponding treatments. The expression level of YAP was significantly lower after VP or HANP/VP treatment compared with the control group, with or without laser irradiation (n = 3/group). **(B)** Immunofluorescence staining of Lamin A/C was performed to observe changes in nuclear morphology after corresponding treatments. The nuclear membranes of the cells were significantly deformed after HANP/VP treatment with or without laser irradiation (n = 3/group). **(C)** Immunofluorescence staining of γH2AX was performed to evaluate nDNA damage after corresponding treatments. Signals of nDNA damage were observed after HANP/VP treatment with or without laser irradiation (n = 3/group). **(D)** Mitotracker staining was performed to assess mitochondrial morphology after corresponding treatments. Mitochondrial membrane morphology was not affected by the HANP/VP, but was disrupted after HANP/VP combined laser irradiation treatment. Laser alone did not affect the mitochondrial membrane morphology (n = 3/group). **(E)** Changes in mitochondrial membrane potential were assessed by JC-1 staining after corresponding treatments. After HANP/VP combined laser irradiation treatment, JC-1 was mostly present as a monomer (green) and the mitochondrial membrane potential decreased (n = 3/group). **(F)** qPCR was used to determine cytosolic mtDNA levels after corresponding treatments. HANP/VP combined laser irradiation treatment increased cytosolic *D-loop* and *ND1* levels (n = 3/group). Statistical significance in **(A)**, **(C)**, and **(F)** was determined by one-way ANOVA (∗∗*p* < 0.01, ∗∗∗*p* < 0.001).

Targeting YAP has emerged as a promising strategy to reverse immunosuppression and sensitize UM tumors to immunotherapy [58]. First, HANP/VP was produced and characterized using the same approach as we previously reported (Fig. S1) [33]. We then evaluated the effect of HANP/VP on YAP expression in the mouse melanoma cell line B16F10 using a Western blot (WB) assay. Cells were treated with free VP (1 μM) and HANP/VP (VP-equivalent dose of 1 μM) for 12 h. A subset received laser (690 nm, 100 mW/cm^2^, 5 min). Cells with and without laser were used as controls. As Fig. 1A shows, HANP/VP remarkably reduced YAP levels by about 70 % compared with untreated cells, while free VP downregulated YAP by 30 % regardless of laser, indicating that HANP/VP more potently inhibited YAP due to improved biocompatibility and tumor cell targetability. The laser alone did not affect YAP expression in B16F10 cells compared with untreated cells, implying that YAP expression was affected by VP or HANP/VP but not by laser.

PDT, initially developed as a minimally invasive laser treatment for retinal vascular diseases [59], generates reactive oxygen species (ROS) that induce double-strand DNA breaks and activate the DNA damage response in both nuclear and mitochondrial genomes, leading to repair, cell cycle arrest, or apoptosis [3,60]. Although PDT is not a first-line treatment for most systemic cancers, it is clinically established for superficial or localized malignancies, particularly those accessible to light penetration such as UM, while minimizing collateral damage, thereby preserving vision. It has also been reported to induce ICD, which can further enhance immunotherapy when used in combination with immune checkpoint inhibitors (ICIs) [61,62].

To assess whether HANP/VP induced nDNA damage in B16F10 cells, we first examined nuclear-membrane integrity via immunofluorescent (IF) staining of Lamin A/C, a key structural component of the nuclear lamina responsible for maintaining nuclear architecture (Fig. 1B). Treatment of B16F10 cells with HANP/VP alone induced marked nuclear abnormalities, including wrinkling, invaginations, and lobulation, consistent with nuclear destabilization caused by YAP inhibition (Fig. 1B). After laser iradiation (Fig. 1B), these defects were further exacerbated, with cells exhibiting pronounced membrane disruption and irregularly shaped nuclear envelopes, indicative of aggravated nuclear instability, supporting that PDT and YAP inhibition by HANP/VP alone induced two routes of DNA damage. Conversely, HANP/VP did not affect nuclear-membrane morphology in the normal human retinal-pigment epithelial (RPE) cell line with or without laser, which proved it to be safe for normal cells in the eye (Fig. S3). Subsequently, we evaluated nDNA damage via γ–H2A histone family member X (γH2AX) staining (Fig. 1C). No obvious γH2AX fluorescence signal was detected in the untreated control group (1.04 ± 1.65 a.u.), but γH2AX signals were markedly increased in cells that received laser (5731.03 ± 1015.18 a.u.) as well as cells that did not diation (3234.61 ± 231.45 a.u.), in the presence of HANP/VP.

Next, we assessed the effect of PDT on mitochondrial integrity. In comparison with the untreated control group, HANP/VP alone did not affect mitochondrial-membrane morphology according to MitoTracker staining. When HANP/VP was combined with laser, we observed severe mitochondrial fragmentation and loss of tubular morphology, with mitochondria appearing punctate and rounded (Fig. 1D), indicative of structural compromise. To further evaluate mitochondrial function, we measured mitochondrial-membrane potential (ΔΨ_m_) using tetraethylbenzimidazolylcarbocyanine iodide (JC-1) staining (Fig. 1E). In the HANP/VP + Laser group, JC-1 was mainly present in its monomeric form, producing strong green fluorescence and reflecting a pronounced loss of ΔΨ_m_ consistent with mitochondrial dysfunction. By contrast, both the untreated-control and HANP/VP groups without laser mainly exhibited JC-1 aggregates, indicating preserved mitochondrial function. In normal ARPE-19 cells, HANP/VP treatment alone did not affect mitochondrial morphology (Fig. S4A), and JC-1 staining showed a predominance of aggregate forms (Fig. S4B), confirming normal ΔΨ_m_ and intact mitochondrial function. Collectively, these findings demonstrated that HANP/VP inhibition of YAP alone did not affect mitochondrial function, but HANP/VP + Laser selectively disrupted mitochondrial structure and function inB16F10 cells while sparing normal retinal cells. Indeed, when ARPE-19 cells were incubated with HANP/VP at different concentrations for different durations, cytotoxic testing indicated no inhibition of normal-cell proliferation (Fig. S5). HANP/VP shows no obvious toxicity on RPE cells, due to the low expression of CD44 receptors in these quiescent cells [63]. In contrast, CD44 is highly expressed in UM and many other tumor types [64,65], leading to preferential uptake and strong cytotoxicity of HANP/VP in tumor cells. Further studies are warranted to elucidate the mechanisms underlying this target specificity and the differential HA receptor expression between tumor and RPE cells.

To assess whether mitochondrial damage resulted in mtDNA leakage, we then quantified cytosolic mtDNA (reduced nicotinamide adenine dinucleotide dehydrogenase 1 [ND1] and D-loop regions) in B16F10 cells (Fig. 1F). Compared with untreated controls, HANP/VP + Laser markedly increased cytoplasmic ND1 and D-loop levels by 2.58 ± 0.04- and 3.60 ± 0.08-fold, respectively, whereas HANP/VP alone showed no obvious changes, suggesting that HANP/VP–mediated YAP inhibition primarily causes nDNA damage but does not induce damage to mtDNA. These data showed that HANP/VP exerted combined YAP-inhibitory and PDT effects in UM cells, leading to dual DNA damage in both nDNA and mtDNA.

### 2.2 HANP/VP induced mtDNA and nDNA damage by activating STING in vitro to enhance innate tumor immunity

DNA damage and cytosolic-DNA leakage can activate the cGAS–STING pathway, triggering type I interferon responses and priming DCs and CD8^+^ T cells [66,67]; this converts immunologically “cold” tumors into “hot” ones, thereby enhancing sensitivity to ICIs [68]. While YAP-mediated cGAS–STING activation has been implicated in other cancers, its role in UM remains largely unexplored. Given that YAP is essential to maintaining nuclear-envelope integrity [[69], [70], [71]] and PDT induces nDNA and mtDNA damage, combining YAP inhibition with PDT provides a unique opportunity to synergistically activate the cGAS–STING pathway, remodel the TME, and boost antitumor immunity.

**Fig. 2:**
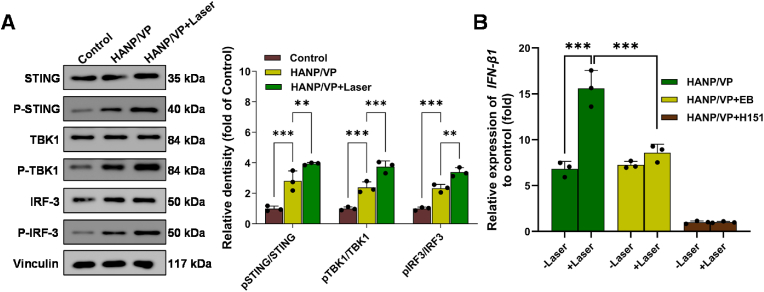
**Activation of the cGAS-STING pathway by HANP/VP via YAP-inhibition and PDT in vitro. (A)** The phosphorylation levels of representative proteins of the cGAS-STING pathway were analyzed using Western blot (*left*) and quantified (*right*). After HANP/VP treatment without laser irradiation, the pSTING/STING, pTBK1/TBK1, and pIRF3/IRF3 ratio were significantly increased compared to the untreated cells; the phosphorylation levels were further increased after laser irradiation, n = 3/group. **(B)** qPCR was used to detect IFN-β1 mRNA levels. Compared with the control group, HANP/VP without Laser treatment significantly increased IFN-β1 expression in B16F10 cells, while HANP/VP combined with laser irradiation further elevated IFN-β1 mRNA levels. When cells were pretreated with ethidium bromide (EB, 120 ng/mL), IFN-β1 mRNA levels decreased in the HANP/VP with Laser irradiation group. Following H151 treatment, IFN-β1 mRNA levels decreased to control group levels regardless of laser irradiation status. n = 3/group. Statistical significance was determined by one-way ANOVA (∗∗*p* < 0.01, ∗∗∗*p* < 0.001).

To confirm activation of the cGAS–STING pathway by HANP/VP–induced dual DNA damage in B16F10 cells, we examined the expression and phosphorylation of key proteins in this pathway via WB. Total STING levels did not change significantly, but our results indicated that HANP/VP markedly enhanced STING phosphorylation and subsequently stimulated downstream TBK1 and IRF3 phosphorylation in B16F10 cells both with and without laser. Notably, levels of phosphorylated STING (p-STING), TBK1 (*p*-TBK1), and IRF3 (*p*-IRF3) were markedly elevated in the HANP/VP + Laser group relative to the HANP/VP–alone group (Fig. 2A). After HANP/VP + Laser treatment, p-STING/STING, *p*-TBK1/TBK1, and *p*-IRF3/IRF3 ratios increased 3.95 ± 0.08-, 3.75 ± 0.38-, and 3.39 ± 0.29-fold compared with untreated cells, respectively, but in cells treated with HANP/VP alone these rations merely increased 2.81 ± 0.66-, 2.38 ± 0.37-, and 2.33 ± 0.25-fold (Fig. 2A). *IFN-β*, a crucial component of the body's defense against intracellular pathogens and cancer, is released when stimulated by STING [72]. Reverse transcription quantitative polymerase chain reaction (RT-qPCR) analysis consistently showed that HANP/VP alone significantly increased expression of *IFN-β1* messenger ribonucleic acid (mRNA) to 6.82 ± 0.81-fold of that of the control group; such expression further increased to 15.60 ± 1.97-fold after laser (Fig. 2B). However, when mtDNA was depleted by ethidium bromide (Fig. S6), *IFN-β1* levels in the HANP/VP + Laser group increased only modestly compared with the control group (8.59 ± 0.93-fold), while slightly lower *IFN-β1* was detected after treatment with HANP/VP alone (7.24 ± 0.40-fold). This implied that nDNA and mtDNA damage induced stronger STING action and immune response (Fig. 2B). To further confirm that HANP/VP enhanced tumor immunity by activating STING, a selective and covalent STING antagonist, H151, was used. We noted that H151 dramatically decreased *IFN-β1* to levels similar to those of the control group, whether or not laser was used (Fig. 2B). Our findings revealed that HANP/VP activated the cGAS–STING pathway primarily through YAP inhibition–mediated nDNA damage in the absence of irradiation, while laser induced both nDNA and mtDNA damage. This dual mechanism provided multiple routes to amplifying cGAS–STING signaling, thereby strengthening innate immune activation and reducing therapeutic resistance. Although the use of the pharmacological inhibitor VP strongly supports the role of YAP inhibition in initiating this cascade, future studies employing YAP knockout models could provide additional genetic validation.

### 2.3 HANP/VP induced ICD via PDT to enhance adaptive tumor immunity

**Fig. 3:**
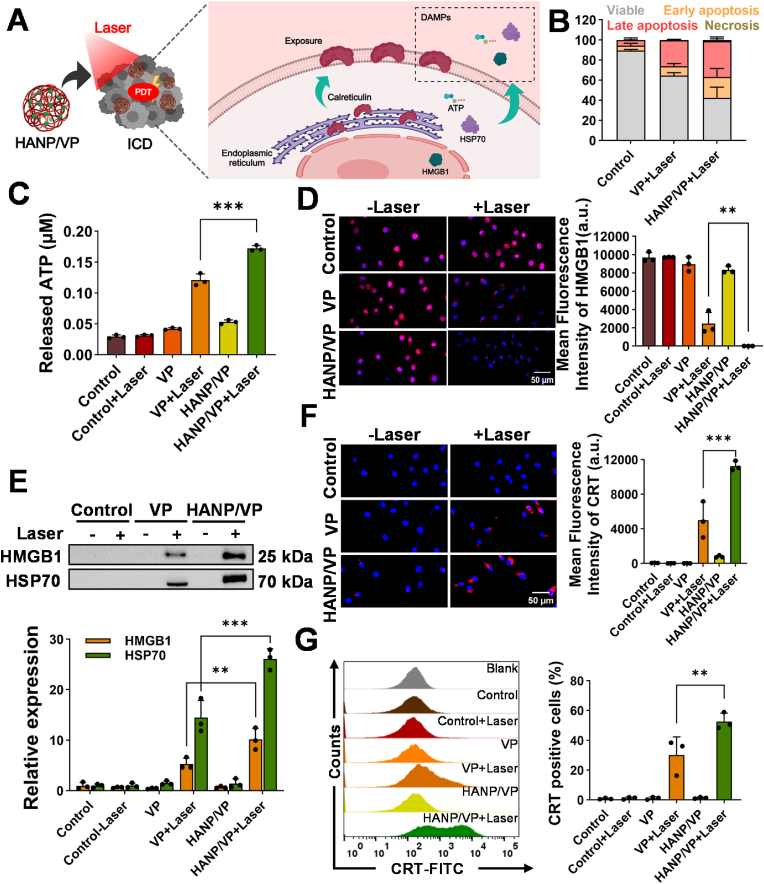
**HANP/VP induces ICD via PDT in vitro. (A)** Schematic illustration of ICD induction by HANP/VP. n = 3/group. **(B)** Apoptosis was analyzed by Annxin V/PI staining after corresponding treatments. After HANP/VP combined laser irradiation treatment, the proportions of early apoptosis and late apoptosis were much higher than those in the free VP combined laser irradiation-treated group, n = 3/group. **(C)** Chemiluminescence was used to detect ATP release after corresponding treatments. After HANP/VP combined laser irradiation treatment, the extracellular ATP concentration was much higher than that in the VP combined laser irradiation treatment group, n = 3/group. **(D)** Immunofluorescence staining was performed to assess the expression of HMGB1 (purple) after corresponding treatments. The level of nuclear HMGB1 expression was reduced after HANP/VP combined laser irradiation, n = 3/group. **(E)** The expression level of HMGB1 and HSP70 in cell culture supernatants was detected by Western blot after corresponding treatments. Compared with the VP with Laser group, the levels of HMGB1 and HSP70 in the cell culture supernatant were much higher after HANP/VP combined laser irradiation, n = 3/group. **(F)** Immunofluorescence staining was used to detect ecto-CRT expression (purple) after corresponding treatments. After HANP/VP combined laser irradiation, significant ecto-CRT signals appeared at a higher level than the VP combined laser irradiation treatment, and no ecto-CRT signals were observed in the control group, n = 3/group. **(G)** The proportion of ecto-CRT-positive cells was analyzed by flow cytometry after corresponding treatments. After HANP/VP combined laser irradiation treatment, the proportion of ecto-CRT-positive cells was significantly higher than that in the VP combined laser irradiation treatment group (30.23 ± 12.13 %), and there were almost no ecto-CRT-positive cells in the Control group, n = 3/group. Statistical significance in panels **(C**–**G)** was determined by one-way ANOVA (∗∗*p* < 0.01, ∗∗∗*p* < 0.001).

PDT not only kills tumor cells directly through ROS generation but also induces ICD [49,73], which promotes DC maturation and T-cell activation while improving tumor immunity via release of DAMPs (Fig. 3A). We next assessed ICD by examining DAMP expression in B16F10 cells following HANP/VP + Laser treatment. First, apoptosis in B16F10 cells was assessed using Annexin V/propidium iodide (PI) staining to determine the mode of cell death induced by HANP/VP under 690-nm laser (100 mW/cm^2^, 5 min; Fig. 3B–S7). Free VP induced early and late apoptosis in respectively 9.31 ± 2.76 % and 25.77 ± 1.20 % of cells, while HANP/VP markedly increased the percentage of early apoptotic cells to 20.83 ± 8.34 % and that of late apoptotic cells to 35.53 ± 4.23 % (Fig. 3B). Since early apoptosis is associated with externalized-CRT (ecto-CRT) exposure and late apoptosis with HMGB1 and ATP release [74], HANP/VP–mediated PDT was expected to initiate stronger ICD to enhance tumor immunogenicity. Indeed, free VP + Laser slightly elevated extracellular-ATP levels to 0.12 ± 0.01 μM compared with 0.03 ± 0.003 μM in untreated controls and 0.03 ± 0.002 μM in cells treated with laser alone, but HANP/VP + Laser dramatically increased this value to 0.17 ± 0.004 μM (Fig. 3C). HANP/VP + Laser treatment was found to decrease nuclear-HMGB1 expression by 1516.81- and 383.58-fold compared with no treatment and VP + Laser treatment, respectively. Laser or VP alone did not change HMGB1 levels, indicating that nuclear HMGB1 was translocated to cytoplasm and then released into the extracellular space under stronger PDT (Fig. 3D–S8). To quantify the released HMGB1, we detected HMGB1 and HSP70 release in culture supernatants via WB (Fig. 3E). Our results showed that HMGB1 increased by 10.16 ± 2.16-fold and HSP70 by 26.08 ± 2.03-fold compared with untreated cells. In contrast, VP + Laser induced modest changes in HMGB1 and HSP70, 5.29 ± 1.17- and 14.44 ± 3.40-fold, respectively (Fig. 3E). Laser alone, free VP alone, and HANP/VP alone did not affect the release of HMGB1 or HSP70 in our study.

Ecto-CRT is an “eat me” protein signal on the surface of dying cells that triggers the immune system to clear these cells, particularly in the context of ICD [75]. After HANP/VP + Laser treatment, a high level of ecto-CRT was detected on B16F10 cells, while only a slight increase was observed after free VP + Laser treatment (Fig. 3F). These changes in ecto-CRT were confirmed via flow cytometry (FCM; BD FACSCalibur; BD Biosciences, Franklin Lakes, NJ, USA), which showed that HANP/VP + Laser treatment elevated ecto-CRT by 52.53 ± 5.61 % compared with untreated control cells, significantly higher than VP + Laser (30.23 ± 12.13 %; Fig. 3G). Taken together, these findings demonstrated that HANP/VP–mediated PDT induced strong ICD in B16F10 cells, characterized by apoptosis and the release of ATP, HMGB1, HSP70, and ecto-CRT. This strategy establishes a strong foundation for stimulating adaptive antitumor immunity and potentially improving the efficacy of immunotherapy in UM. While PDT offers a unique dual mechanism of direct tumor cytotoxicity and ICD, its clinical application may be limited in certain contexts, such as UM liver metastases, where laser delivery is not feasible. In these scenarios, HANP/VP-mediated YAP inhibition remains a potent strategy to reprogram the tumor microenvironment. Importantly, combining HANP/VP with ICB has demonstrated the capacity to elicit robust antitumor immune responses in preclinical models, underscoring its potential to treat metastatic disease beyond the reach of PDT. Future studies will focus on developing an advanced in vitro co-culture model to recapitulate the entire immune cascade, from tumor cell death to the activation of tumor-specific T lymphocytes, thereby strengthening the mechanistic basis of this combinatorial therapeutic strategy.

### 2.4 HANP/VP inhibited tumor growth and prolonged survival in a UM tumor mouse model

Due to the lack of a syngeneic mouse UM model, we employed the well-established B16F10 syngeneic model inoculated into the subretinal space as the optimal surrogate [[76], [77], [78]]. Because UM arises in the unique ocular microenvironment, its TME differs substantially from that of ectopic tumors with distinct immune privilege, stromal composition, and vascular characteristics that strongly influence therapeutic response. The use of orthotopic UM models provides critical advantages for evaluating immunotherapeutic strategies over conventional subcutaneous models. This approach allowed us to investigate the conserved mechanisms of anti-tumor immunity within a fully immunocompetent microenvironment, thereby providing critical insights into the immuno-activatory potential of our strategy. Importantly, the orthotopic model allows assessment of how HANPs and immunotherapies perform in the eye's immune-privileged site, where mechanisms of immune evasion are particularly pronounced. Incorporating orthotopic models, therefore, not only captures the physiological context of UM growth and immune suppression but also enables realistic evaluation of therapeutic efficacy, safety, and translational potential for vision-preserving HANP-based immunotherapy.

**Fig. 4:**
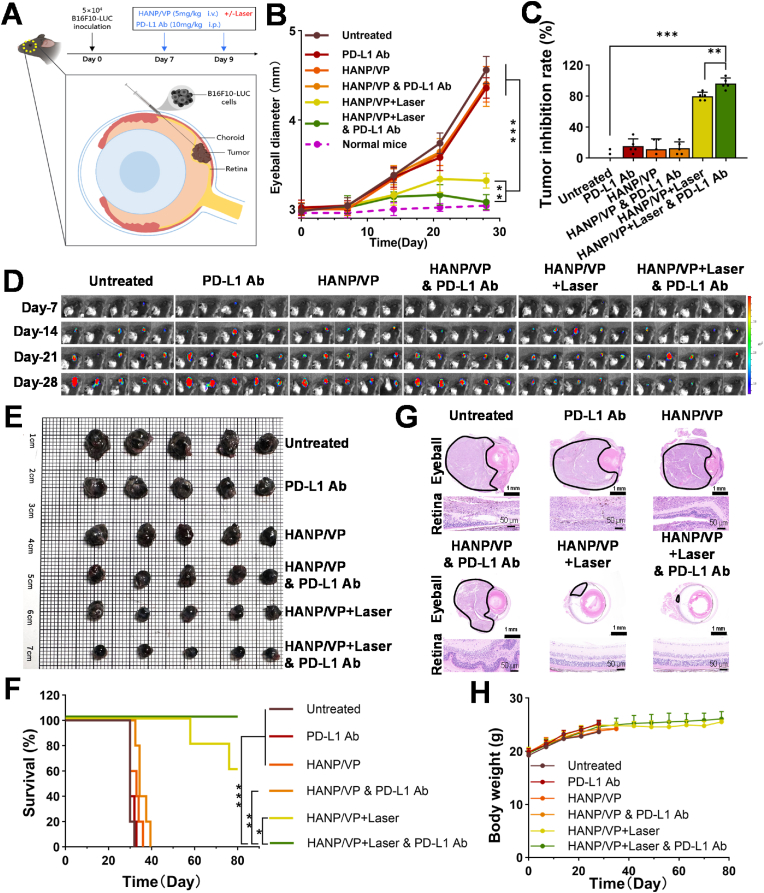
**In vivo antitumor effects of HANP/VP. (A)** Schematic illustration of the establishment and treatment schedule of the orthotopic UM mice models. **(B–C)** Eyeball diameter growth curves **(B)** and tumor inhibition rate **(C)** following various treatments in the orthotopic UM mice models. The combination of mouse PD-L1 Ab with HANP/VP and laser illumination significantly prevents tumor growth. (n = 5 mice/group). **(D)** IVIS imaging showing tumor growth status post-treatment in the orthotopic UM mice models (n = 5 mice/group). **(E)** Gross images of excised eyeballs from each group on day 28 after tumor cell inoculation (n = 5 mice/group). **(F)** Survival curves of mice in each treatment group. 60 % of mice treated with HANP/VP and laser survived up to day 80, while 100 % mice receiving the HANP/VP and laser combined with the PD-L1 Ab combination survived to day 80 (n = 5 mice/group). **(G)** Representative H&E staining images of ocular and retinal structures from each group, with tumor regions indicated by black lines (n = 5 mice/group). **(H)** Body weight changes in each group during the treatment period (n = 5 mice/group). Statistical significance in **(B–C)** was determined by two-way ANOVA; **(F)** was determined by log-rank test (∗*p* < 0.05, ∗∗*p* < 0.01, ∗∗∗*p* < 0.001).

In this study, an orthotopic UM model was established by injecting luciferase-transfected B16F10 cells into mouse eyeballs and performing two treatment sessions, on days 7 and 9 after tumor cell inoculation (Fig. 4A). Eyeball diameter was measured and recorded (Fig. 4B). UM tumors grew slowly during week 1 and then started to grow larger in the untreated, PD-L1 Ab–, HANP/VP–, and HANP/VP & PD-L1 Ab–treated mouse groups. Although HANP/VP + Laser slowed tumor growth in mice, HANP/VP + Laser & PD-L1 Ab inhibited such growth more significantly (Fig. 4B), by almost 96.20 ± 7.22 % (Fig. 4C). However, tumor growth was not remarkably affected when HANP/VP was combined with mouse PD-L1 Ab without laser.

We also used bioluminescence signals to monitor development of UM tumors. As shown in Fig. 4D, these signals increased in the control groups but almost disappeared in the groups receiving HANP/VP + Laser & PD-L1 Ab. After 4 weeks, mice were sacrificed, and the eyeballs containing UM were collected. Gross images (Fig. 4E) and volumetric measurements (Fig. S9) of excised eyeballs showed that HANP/VP + Laser & PD-L1 Ab group exhibited significantly smaller eyeball volume (14.29 ± 1.38 mm^3^) compared to the untreated group (49.40 ± 4.54 mm^3^) and HANP/VP + Laser group (21.94 ± 0.73 mm^3^). Hematoxylin and eosin (H&E) staining showed that mouse eyeballs were restored to normal after HANP/VP + Laser & PD-L1 Ab treatment (Fig. 4G). HANP/VP + Laser also modestly inhibited tumor growth, but the inhibition rate could be improved by adding PD-L1 Ab. Free PD-L1 Ab and HANP/VP did not significantly inhibit tumor growth regardless of laser , as observed in H&E-stained eyeballs (Fig. 4G). More importantly, eyes from the HANP/VP + Laser group and the HANP/VP + Laser & PD-L1 Ab groups had largely preserved ocular architecture with clearly discernible internal structures. In contrast, marked tumor invasion was observed in the other groups, often obliterating the vitreous chamber and penetrating the outer ocular layers. Notably, in the untreated and PD-L1 Ab–treated groups, aggressive tumor proliferation rendered the retina nearly indistinguishable, reflecting severe disruption of ocular integrity (Fig. 4G).

To further assess whether improved therapeutic efficacy translated into durable survival benefits, we conducted a survival study in UM-bearing mice. Animals were euthanized on reaching humane endpoints, including ocular rupture, corneal ulceration, or inability to consume food or water. All mice in the untreated group succumbed by day 32, while those in the PD-L1 Ab– and HANP/VP–treated groups reached endpoints by days 33 and 36, respectively. In the HANP/VP & PD-L1 Ab group, 20 % of mice survived to day 37, but 60 % of mice treated with HANP/VP + Laser survived to day 80. All of the mice receiving HANP/VP + Laser & PD-L1 Ab survived to day 80 (Fig. 4F). These results demonstrated that HANP/VP–mediated PDT could significantly prolong survival and achieved the most pronounced benefit when combined with PD-L1 blockade, indicating great potency for clinically treating UM. No significant changes in body weight (BW) were observed across treatment groups (Fig. 4H). We also assessed retinal function in mice using flash electroretinography (F-ERG) (Fig. S10A). Results showed that retinal function was significantly impaired in the untreated group due to rapid tumor growth, with complete loss of electrophysiological signals. In contrast, the HANP/VP + Laser group exhibited normal characteristics of the a-wave (reflecting photoreceptor cell activity) and b-wave (reflecting bipolar cell activity), similar to normal mice, indicating preserved retinal function. Furthermore, H&E staining of key mouse organs revealed no significant histological alterations in any normal organs following HANP/VP treatment (Fig. S10B). These results support the biosafety and systemic tolerability of HANP/VP.

### 2.5 *In vivo* validation of therapeutic response to HANP/VP & PD-L1 Ab treatment in UM

**Fig. 5:**
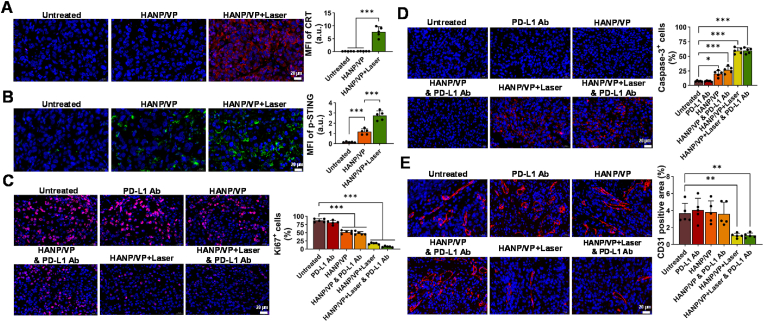
**HANP/VP activates the cGAS-STING pathway and ICD in vivo, inhibits tumor proliferation, and disrupts tumor vasculature. (A)** CRT staining (red) to evaluate intratumoral ICD (n = 5 mice/group). **(B)** p-STING staining (green) to assess activation of the cGAS–STING pathway in tumor tissues (n = 5 mice/group). **(C)** Ki67 staining (red) to assess tumor cell proliferation. Following HANP/VP and laser combined with PD-L1 Ab treatment, Ki67+ cells significantly decreased compared to the untreated control group (n = 5 mice/group). **(D)** Caspase-3 staining (red) to evaluate tumor cell apoptosis. The apoptosis rate induced by HANP/VP and laser (with or without PD-L1 antibody) was significantly higher than that in the untreated, PD-L1 Ab, HANP/VP, and HANP/VP combination with PD-L1 Ab groups (n = 5 mice/group). **(E)** CD31 staining (red) to assess the tumor vasculature. HANP/VP-mediated PDT in combination with or without PD-L1 Ab induced marked vascular disruption compared to the untreated group (n = 5 mice/group). Statistical significance was determined by one-way ANOVA (∗*p* < 0.05, ∗∗*p* < 0.01, ∗∗∗*p* < 0.001).

To examine therapeutic response after different treatments and validate treatment responses, we evaluated intratumoral ICD by ecto-CRT expression (Fig. 5A). No notable ecto-CRT signals were detected in the untreated group (0.12 ± 0.01 a.u.) or the HANP/VP–alone group (0.14 ± 0.02 a.u.), but ecto-CRT was significantly upregulated in the HANP/VP + Laser group (7.64 ± 2.0 a.u.), indicating that HANP/VP–mediated PDT effectively induced ICD in tumor tissues. STING activation was also assessed via IF staining of p-STING in tumor tissues (Fig. 5B). In comparison with the untreated group (0.16 ± 0.04 a.u.), the HANP/VP–alone group showed a significant elevation in p-STING signal (1.17 ± 0.28 a.u.), which was further amplified in the HANP/VP + Laser group (2.75 ± 0.46 a.u.). This indicated that HANP/VP activated the cGAS–STING pathway *in vivo* by both YAP inhibition and PDT. STING pathway activation induced by dual DNA damage could broaden the application of HANP/VP to treat other types of tumors when PDT is not feasible.

Next, cell proliferation was examined using antigen Kiel 67 (Ki-67) immunostaining (Fig. 5C). We observed a remarkable reduction, to 7.33 ± 1.80 %, in Ki-67^+^ cells treated with HANP/VP + Laser & PD-L1 Ab, compared with 16.98 ± 2.59 % in the HANP/VP + Laser group, 51.77 ± 5.36 % in the HANP/VP–alone group, 48.26 ± 4.93 % in the HANP/VP & PD-L1 Ab without laser group, and 87.14 ± 5.23 % in the untreated group. Meanwhile, apoptotic levels were evaluated using active cysteine–aspartic acid–specific protease/proteinase-3 (Caspase-3) IF staining (Fig. 5D). HANP/VP + Laser with and without PD-L1 Ab elicited apoptosis rates of 59.77 ± 4.89 % and 59.66 ± 5.23 %, respectively, which significantly surpassed those of the untreated (7.32 ± 0.92 %), PD-L1 Ab (7.39 ± 0.85 %), HANP/VP–alone (14.91 ± 3.30 %), and HANP/VP & PD-L1 Ab (19.18 ± 5.69 %) groups. This emphasized that HANP/VP–mediated PDT was critical to inducing apoptosis in UM cells. PDT has been reported to reduce CD31^+^ blood vessels via ROS-mediated vascular damage [79], leading to impaired perfusion and nutrient supply. In UM, where angiogenesis drives tumor growth and immune evasion, we observed no blood vessel changes in the untreated (3.66 ± 1.17 %), PD-L1 Ab (4.05 ± 1.37 %), HANP/VP (3.80 ± 1.31 %), or HANP/VP & PD-L1 Ab (3.60 ± 1.37 %) groups. In contrast, HANP/VP–mediated PDT with or without PD-L1 Ab induced vascular disruption (1.07 ± 0.29 % and 1.05 ± 0.27 %, respectively; Fig. 5E), which were decreased by 3.42- and 3.49-fold compared with the untreated group, indicating the YAP inhibition and PDT not only suppressed tumor progression but also possibly reshaped the TME to enhance immune cell infiltration and improve immunotherapeutic response.

### 2.6 Immune responses of UM tumor after HANP/VP–mediated YAP-inhibition/PDT treatment

**Fig. 6:**
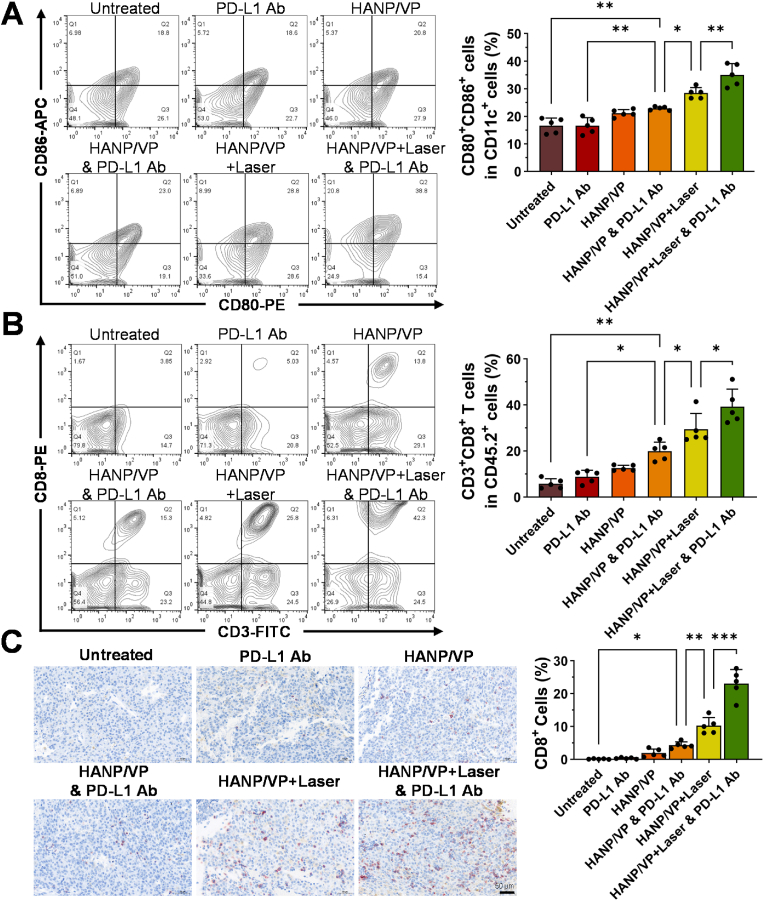
**HANP/VP enhances intratumoral immune infiltration and induces antitumor immune responses. (A)** Flow cytometry analysis of the percentage of CD80^+^CD86^+^ cells among CD11c^+^ DCs in mouse spleens. Compared to the untreated group, the mature DCs significantly increased in the HANP/VP combined with PD-L1 Ab, and the HANP/VP with laser groups, the HANP/VP with laser combined with PD-L1 Ab treatment maximizes the mature DCs, n = 5 mice/group. **(B)** Flow cytometry analysis of the percentage of CD3^+^CD8^+^ cytotoxic T cells among CD45.2^+^ immune cells in mouse tumor tissues. HANP/VP and laser combined with PD-L1 Ab treatment presented the highest CD8^+^ T cells compared to the untreated group, n = 5 mice/group. **(C)** Immunohistochemical staining of CD8^+^ T cell infiltration in tumor sections. HANP/VP and laser combined with PD-L1 Ab treatment presented the highest CD8^+^ T cells compared to the untreated group, n = 5 mice/group. Statistical significance in **(A**–**C)** was determined by one-way ANOVA (∗*p* < 0.05, ∗∗*p* < 0.01, ∗∗∗*p* < 0.001).

To examine immune responses in UM after different treatments, we assessed changes in CD80^+^CD86^+^CD11c^+^ DCs [80,81] in the spleen and CD3^+^CD8^+^ active T cells in tumors using FCM. Mature DCs increased slightly in the HANP/VP–alone group (21.14 ± 1.33 %) relative to the untreated group (16.66 ± 2.73 %). DCs were further increased in the HANP/VP & PD-L1 Ab group to 22.86 ± 0.64 %, while in the HANP/VP + Laser group this figure rose to 28.46 ± 2.02 % of DCs in splenocytes. The HANP/VP + Laser & PD-L1 Ab group maximized DCs to 35.06 ± 4.04 % (Fig. 6A). These results suggested that HANP/VP promoted DC maturation via YAP inhibition–mediated activation of the cGAS–STING pathway even without laser irradiation, while HANP/VP–induced PDT further amplified this effect by inducing ICD and strengthening STING pathway activation.

Next, infiltration of CD8^+^ T cells into tumor tissue was quantified using FCM. We found that HANP/VP + Laser & PD-L1 Ab treatment yielded the highest CD8^+^ T-cell count (39.30 ± 7.59 %) in UM tumors, compared with the untreated (5.85 ± 2.17 %), PD-L1 Ab–alone (8.89 ± 2.73 %), HANP/VP–alone (12.64 ± 1.17 %), HANP/VP + Laser (29.44 ± 6.85 %), and HANP/VP & PD-L1 Ab (19.92 ± 3.97 %) groups (Fig. 6B). CD8^+^ T-cell changes were also consistent with the tumor immunohistochemistry of CD8^+^ T cells (Fig. 6C). HANP/VP + Laser & PD-L1 Ab treatment again yielded the highest CD8^+^ T-cell count (23.07 ± 4.29 %) in UM tumors, compared with the untreated (0.15 ± 0.12 %), PD-L1 Ab–alone (0.36 ± 0.22 %), HANP/VP–alone (1.98 ± 1.22 %), HANP/VP + Laser (10.29 ± 2.50 %), and HANP/VP & PD-L1 Ab (4.35 ± 1.01 %) groups. These observed trends closely aligned with the patterns of mature DCs, supporting the notion that HANP/VP–mediated YAP inhibition + PDT could effectively elicit strong antitumor immune responses.

**Fig. 7:**
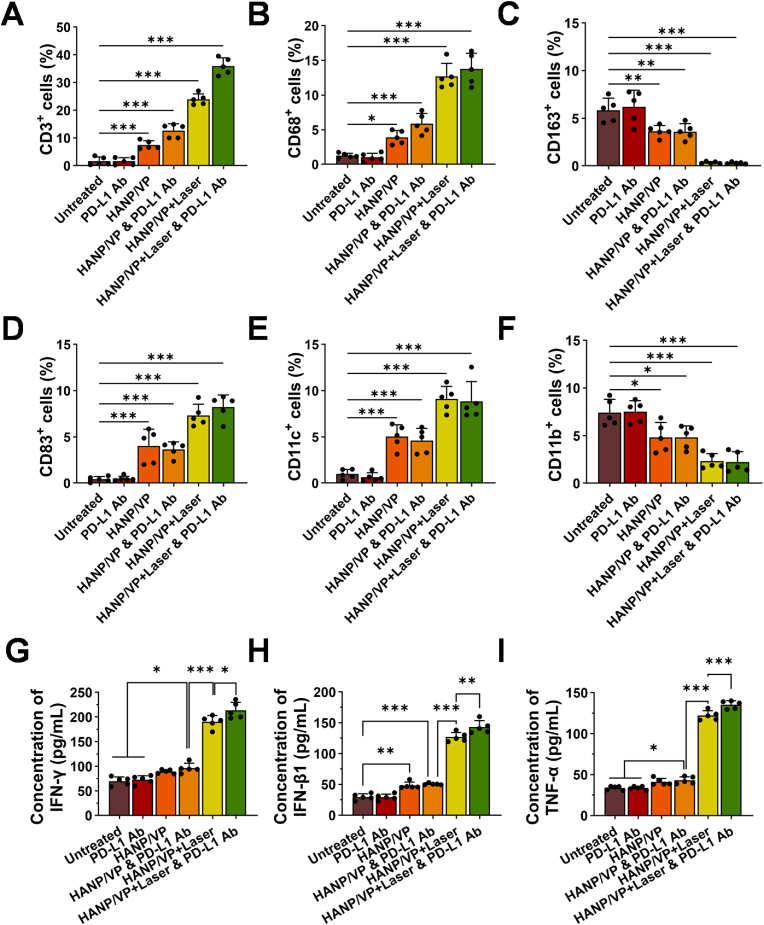
**HANP/VP remodels immune cell populations within the TME. (A)** CD3 staining to analyze T cell infiltration levels in tumor tissues. The ratio of CD3^+^ cells in HANP/VP and laser combined with PD-L1 Ab, HANP/VP and Laser, HANP/VP combined with PD-L1 Ab, and HANP/VP groups were significantly higher than untreated group, n = 5 mice/group. **(B)** CD68 staining to analyze M1 macrophage infiltration levels in tumor tissues. The ratio of CD68^+^ cells in HANP/VP and laser combined with PD-L1 Ab, HANP/VP and Laser, HANP/VP combined with PD-L1 Ab, and HANP/VP groups were significantly higher than untreated group, n = 5 mice/group. **(C)** CD163 staining to analyze M2 macrophage infiltration levels in tumor tissues. **(D)** CD83 staining to analyze infiltration levels of mature DCs in tumor tissues. **(E)** CD11c staining to analyze DCs infiltration levels in tumor tissues. n = 5 mice/group**. (F)** CD11b staining to analyze infiltration levels of myeloid-derived cells in tumor tissues. n = 5 mice/group. **(G)** ELISA to measure serum levels of IFN-γ in each group of mice. n = 5 mice/group. **(H)** ELISA to measure serum levels of IFN-β1 in each group of mice. n = 5 mice/group. **(I)** ELISA to measure serum levels of TNF-α in each group of mice. n = 5 mice/group. Statistical significance was determined by one-way ANOVA (∗*p* < 0.05, ∗∗*p* < 0.01, ∗∗∗*p* < 0.001).

To further investigate the effect of HANP/VP treatment on the TME in UM, we also analyzed immune active cells via IF staining (Fig. 7A–F, S11), including total T-cell (CD3^+^), macrophage (CD68^+^), M2-type macrophage (CD163^+^), and DC (CD83^+^) counts. We observed that HANP/VP + Laser & PD-L1 Ab treatment resulted in the strongest immune response across all groups, with 35.90 ± 3.03 % of total CD3^+^ T cells, 13.76 ± 2.27 % of CD68^+^ M1 macrophages, 0.30 ± 0.10 % of CD163^+^ macrophages, 8.23 ± 1.30 % of CD83^+^ DCs, and 8.83 ± 2.15 % of CD11c^+^ DCs. Conversely, HANP/VP + Laser & PD-L1 Ab treatment reduced immunosuppressive CD11b^+^ cells from 7.42 ± 1.39 % compared with the untreated group to 2.23 ± 1.08 %. We did not observe that PD-L1 Ab alone affected CD11b^+^ immunosuppressive cells in our study, while HANP/VP + Laser or HANP/VP & PD-L1 Ab modestly reduced counts of immunosuppressive cells (Fig. 7A–F), confirming that PDT and YAP inhibition were necessary to enhance the efficacy of immunotherapy.

Given that HANP/VP exerts dual DNA damage via YAP inhibition and PDT, thereby activating the cGAS–STING pathway and inducing ICD, monitoring cytokine secretion is critical to establishing downstream immune consequences. Measuring cytokine profiles, therefore, provides mechanistic evidence that HANP/VP + Laser enhances innate and adaptive antitumor immunity, while also offering insight into the therapeutic potential and safety of this strategy to overcome immunotherapeutic resistance in UM. As Fig. 7G shows, serum IFN-γ level was significantly elevated in the HANP/VP & PD-L1 Ab group (96.15 ± 10.14 pg/mL) compared with the untreated group (69.86 ± 8.77 pg/mL). When laser was applied, IFN-γ levels increased to 190.23 ± 12.74 pg/mL in the HANP/VP + Laser group and were further elevated to 213.51 ± 16.61 pg/mL in the HANP/VP + Laser & PD-L1 Ab group. Similarly, HANP/VP + Laser & PD-L1 Ab resulted in 143.20 ± 10.43 pg/mL of IFN-β1 and 135.29 ± 4.82 pg/mL of TNF-α, significantly higher levels than in the other groups (Fig. 7H and I). Collectively, these findings supported that HANP/VP activated the cGAS–STING pathway via YAP inhibition even in the absence of laser, thereby reprogramming the immunosuppressive TME in UM. When laser was added, HANP/VP further amplified these effects through PDT-induced ICD and enhanced pathway activation, resulting in strong immune stimulation and remodeling of the UM TME with enhanced tumor immunotherapeutic responses. Nevertheless, the current study focuses on preclinical mechanistic exploration without validation in clinical samples. In future translational studies, evaluating YAP expression and STING pathway activation in tumor tissues from UM patients receiving immunotherapy will be essential for determining the clinical relevance of this mechanism and identifying potential biomarkers for patient stratification. Incorporating clinical specimens will also be critical for validating whether HANP/VP–mediated activation of the STING pathway correlates with therapeutic responses in UM patients.

### 2.7 Anti-UM immune memory effect induced by HANP/VP & PD-L1 Ab combination therapy

**Fig. 8:**
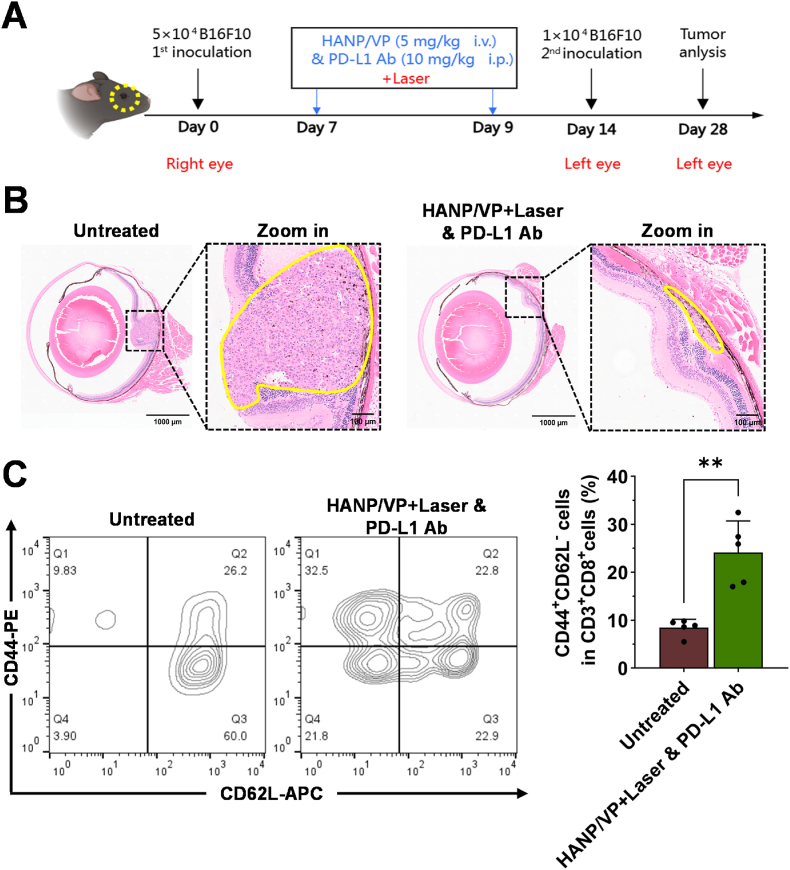
**In vivo antitumor immune memory response induced by HANP/VP & PD-L1 Ab combination therapy. (A)** Schematic illustration of the UM rechallenge models and treatment regimen in mice. **(B)** Representative H&E-stained images of rechallenged tumors in each group, with yellow outlines indicating tumor regions (n = 5 mice/group). **(C)** Flow cytometric analysis of the percentage of CD8^+^ Tem (CD3^+^CD8^+^CD44^+^CD62L^low^) cells in the spleens of mice. n = 5 mice/group. Statistical significance in **(C)** was determined by Student's t-test (∗∗∗*p* < 0.001).

To further evaluate the strategy's therapeutic potential, we examined whether HANP/VP + Laser & PD-L1 Ab could elicit an anti-UM immunological-memory response in a tumor rechallenge model. UM tumor–bearing mice received two treatments as described above and then another UM tumor cell inoculation on the other side of the eye 5 days after the last treatment (Fig. 8A). Mice were sacrificed within 28 days to avoid a large tumor burden. Nevertheless, control mice that received no treatments were still found to have fast tumor growth when rechallenged with B16F10 cells, while HANP/VP + Laser & PD-L1 Ab dramatically prevented growth of the rechallenged UM tumor as confirmed by the H&E staining (Fig. 8B). Following initial recognition of tumor antigens, effector/memory T cells (Tems) can rapidly identify and eradicate tumor cells expressing the same antigens, thereby preventing recurrence and metastasis [82]. FCM analysis revealed that the percentage of Tems (CD3^+^CD8^+^CD44^+^CD62^low^) in the spleens of the HANP/VP + Laser & PD-L1 Ab group (24.14 ± 6.59 %) was significantly higher than that in the untreated group (8.47 ± 1.72 %; Fig. 8C), indicating robust memory immune activation.

These results demonstrated that HANP/VP combined with PD-L1 Ab not only controlled primary UM growth but also induced a durable, specifically anti-UM immunological-memory response to prevent recurrence. Antigen exposure is closely associated with the differentiation of memory T cells [83]. Specifically, the recruitment of antigen-presenting cells and the sustained release of immunogenic substances can effectively stimulate and sustain the generation of memory T cells, thereby maintaining immunological memory against specific antigens [84]. HANP/VP mediates potent release of immunogenic substances and activation of the immune system via cGAS-STING activation and ICD, thereby effectively inducing the differentiation of memory T cells. Moreover, the combined application of PD-L1 Ab, by blocking immune checkpoints, not only restores the function of effector T cells but, crucially, creates a favourable TME for the differentiation and long-term survival of memory T cells [85]. Multiple studies on nanomedicine immunotherapy indicate that inducing immune memory responses can be achieved through ICD or by activating the STING pathway [86,87]. However, most of these studies rely on subcutaneous tumor models, which exhibit inherently higher baseline T-cell infiltration and immune activation than orthotopic UM. Adopting the orthotopic UM model in our study provides a more clinically relevant environment, particularly for immune “cold” UM.

### 2.8 Conclusion

In this study, we investigated the mechanism of the HANP-based formulation of VP that integrated YAP inhibition with PDT to overcome resistance to immunotherapy in UM. Our results confirmed that HANP/VP effectively activated the cGAS–STING pathway through dual DNA damage induced by YAP inhibition and PDT while also triggering strong ICD, enhancing DC maturation, and reshaping the immunosuppressive TME. This remodeling converted the “cold” UM tumor into an immunologically “hot” one, thereby amplifying antitumor immune responses and synergizing with PD-L1 blockade to suppress tumor progression. Importantly, HANP/VP combined with PD-L1 Ab and laser not only inhibited primary UM growth but also elicited durable antitumor immunological memory, preventing recurrence and metastasis. Overall, these findings highlight HANP/VP as an innovative nanomedicine for improving poor immunotherapeutic response in UM, providing both mechanistic insights and preclinical evidence in support of enhancing innate and adaptive tumor immunity via the above-described combination approaches.

### 2.9 Materials and methods

#### 2.9.1 Materials

VP, Cell Counting Kit-8 (CCK-8), hyaluronidase, H151, and EB were purchased from MedChemExpress (Monmouth Junction, NJ, USA). Collagenase IV and DNase were obtained from GlpBio (Montclair, CA, USA). Fetal bovine serum (FBS) was purchased from Sigma-Aldrich (Burlington, MA, USA). The Annexin V/PI staining kit was obtained from Hangzhou MultiSciences (Lianke) Biotech Co., Ltd. (Hangzhou, China). An ATP assay kit, MitoTracker, and a JC-1 ΔΨ_m_ assay kit were purchased from Beyotime Institute of Biotechnology (Shanghai, China). A TIANamp DNA Kit was obtained from TIANGEN Biotech Co., Ltd. (Beijing, China). Enhanced-chemiluminescence solution, a Total RNA Kit, a Complementary DNA (cDNA) Synthesis Kit, and Universal Blue qPCR SYBR Green Master Mix were purchased from Yeasen Biotechnology Co., Ltd. (Shanghai, China).

#### 2.9.2 Cell culture

The mouse melanoma cell line B16F10 was purchased from the American Type Culture Collection (ATCC; Manassas, VA, USA). Luciferase-transfected B16F10 cells (B16F10-LUC) and the human RPE cell line ARPE-19 were maintained in our laboratory cell repository. B16F10 and B16F10-LUC cells were cultured in Dulbecco's Modified Eagle's Medium (DMEM), while ARPE-19 cells were cultured in DMEM/Ham's F12 medium. All of the media were supplemented with 10 % FBS and 1 % penicillin–streptomycin at 37 °C in a 5 % CO_2_ atmosphere.

#### 2.9.3 Immunofluorescence staining

Cultured cells or tumor tissue sections were incubated overnight at 4 °C with Abs against Lamin A/C (1:500; ZenBio, Beijing, China), γH2AX (1:300; Wuhan Servicebio Technology Co., Ltd., Wuhan, China), HMGB1 (1:300; Proteintech, Shanghai, China), CRT (1:300; Abcam, Cambridge, UK), Ki-67 (1:500; Servicebio), Caspase-3 (1:500; Servicebio), p-STING (1:100; Cell Signaling Technology [CST], Danvers, MA, USA), CD8 (1:500; Servicebio), CD3 (1:500; Servicebio), CD68 (1:300; Servicebio), CD163 (1:300; Servicebio), CD11b (1:300; Servicebio), CD11c (1:300; Servicebio), CD83 (1:300; ABclonal Technology, Wuhan, China), CD31 (1:500; Servicebio), and PD-L1 (1:300; Abcam); then with fluorescein isothiocyanate (FITC) or Alexa Fluor 594–conjugated secondary Abs (Thermo Fisher Scientific, Waltham, MA, USA) for 1 h at 25 °C. Nuclei were stained with DAPI. Fluorescent images were captured under a confocal microscope and analyzed using ImageJ (US National Institutes of Health [NIH], Bethesda, MD, USA).

#### 2.9.4 Assessment of mitochondrial damage

B16F10 and ARPE-19 cells (∼80 % confluence) were incubated with HANP/VP (1 μM equivalent dose of free VP) for 12 h, then either exposed to laser (690 nm, 100 mW/cm^2^, 5 min) or left untreated. Mitochondrial morphology was assessed using MitoTracker staining, while ΔΨ_m_ was evaluated using JC-1 staining.

#### 2.9.5 Quantification of cytosolic mtDNA

B16F10 (∼80 % confluence) were incubated with HANP/VP (1 μM VP) for 12 h, either exposed to laser (690 nm, 100 mW/cm^2^, 5 min) or left untreated, lysed in 1 % nonyl phenoxypolyethoxylethanol (NP-40) on ice for 15 min, and centrifuged (16,000 *g*, 15 min). Then, cytosolic DNA was extracted using the TIANamp DNA Kit. RT-qPCR as well as primers for ND1 and D-loop were employed to quantify mtDNA [88].

Primers:

*ND1*.

Forward: 5′-CTAGCAGAAACAAACCGGGC-3′

Reverse: 5′-CCGGCTGCGTATTCTACGTT-3′

*D-loop*.

Forward: 5′-TCCTCCGTGAAACCAACAA-3′

Reverse: 5′-AGCGAGAAGAGGGGCATT-3′

*18s*.

Forward: 5′-CGGCTACCACATCCAAGGAA-3′

Reverse: 5′-GCTGGAATTACCGCGGCT-3′

#### 2.9.6 Nuclear-morphology and nDNA damage assessment

B16F10 and ARPE-19 cells (∼80 % confluence) were incubated with HANP/VP (1 μM VP) for 12 h, then either exposed to laser (690 nm, 100 mW/cm^2^, 5 min) or left untreated. Next, cells were stained for Lamin A/C and γH2AX, and nuclear morphology and DNA damage were assessed via confocal microscopy.

#### 2.9.7 Mitochondrial-DNA depletion

B16F10 cells were cultured in medium with EB (120 ng/mL) for 3 days. DNA was extracted, and mtDNA depletion was confirmed via RT-qPCR.

#### 2.9.8 RNA extraction, cDNA synthesis, and RT-qPCR

Total RNA was extracted and cDNA synthesized, followed by RT-qPCR. Relative gene expression was calculated using the 2^−ΔΔCt^ method.

Primers:

*IFN-β1*.

Forward: 5′-GCCTTTGCCATCCAAGAGATGC-3′

Reverse: 5′-ACACTGTCTGCTGGTGGAGTTC-3′

*β-actin*.

Forward: 5′-CATTGCTGACAGGATGCAGAAGG-3′

Reverse: 5′-TGCTGGAAGGTGGACAGTGAGG-3′

#### 2.9.9 Western blot analysis

Cells were treated with free VP (1 μM) and HANP/VP (VP-equivalent dose of 1 μM) for 12 h; some then received laser (690 nm, 100 mW/cm^2^, 5 min). Both radiated and non-radiated cells were used as controls. Proteins extracted from cells or cell culture supernatants were separated via sodium dodecyl sulfate polyacrylamide gel electrophoresis (SDS-PAGE) and transferred to polyvinylidene fluoride (PVDF) membranes. The membranes were blocked in 5 % skim milk; incubated overnight at 4 °C with Abs against YAP1 (1:1000; Proteintech), STING (1:2000; CST), p-STING (1:1000; CST), TBK1 (1:2000; CST), *p*-TBK1 (1:1000; CST), IRF-3 (1:2000; CST), *p*-IRF-3 (1:1000; CST), vinculin (1:3000; Proteintech), HMGB1 (1:000; Proteintech), and HSP70 (1:1000; Proteintech); and then incubated with appropriate secondary Abs for 1 h at 25 °C. Signals were visualized using ECL and analyzed using ImageJ.

#### 2.9.10 Cytotoxicity assay

ARPE-19 cells (5 × 10^4^) were seeded into a 96-well plate and treated with HANP/VP at VP-equivalent concentrations of 0.25, 0.5, 1, 2, and 4 μM for 12 h, followed by laser (690 nm, 100 mW/cm^2^, 5 min) or, for controls, a lack thereof. The cells were also treated with 1 μM HANP/VP for 12, 24, and 48 h, followed again by either laser (690 nm, 100 mW/cm^2^, 5 min) or the absence thereof for controls. Finally, cells were incubated with 10 % CCK-8 solution for 1 h, and absorbance was measured at 450 nm using a microplate reader to calculate relative cell viability.

#### 2.9.11 Apoptosis assay

B16F10 cells grown to approximately 80 % confluence were treated with either VP (1 μM) or HANP/VP (VP-equivalent dose of 1 μM) for 12 h followed by laser (690 nm, 100 mW/cm^2^, 5 min). Cells from each group were then digested in Accutase and stained with Annexin V–FITC- and PI-containing buffer for 10 min. Apoptosis was analyzed via FCM.

#### 2.9.12 Assessment of ICD

B16F10 cells at approximately 80 % confluence were incubated with VP (1 μM) or HANP/VP (VP-equivalent dose of 1 μM) for 12 h. Next, some of these cells received laser at 690 nm (100 mW/cm^2^) for 5 min, while others, as controls, did not. Cell culture supernatants were collected, and HMGB1 and HSP70 levels were quantified via WB [61,62]. ATP levels in supernatants were measured using the ATP assay kit. Cells were then incubated with anti-CRT Ab, followed by fluorescent secondary-Ab labeling. Membrane exposure to CRT was analyzed using confocal microscopy and FCM.

#### 2.9.13 Orthotopic UM mouse model

All of the animal experimental procedures were conducted in accordance with the *Guide for the Care and Use of Laboratory Animals* published by the NIH, and all of the protocols were approved by the Animal Welfare and Ethics Committee of Jilin University (Changchun, China). Female C57BL/6 mice (6–8 weeks old, 18–20 g) were purchased from Beijing Vital River Laboratory Animal Technology Co., Ltd. (Beijing, China). Mice were maintained in a pathogen-free environment under controlled temperature, with *ad libitum* access to food and water. To establish the orthotopic UM mouse model, a 32G needle was used to penetrate the eyeball wall just behind the corneoscleral limbus to create an injection channel. Then, a 33G Hamilton microsyringe was used to inject B16F10-LUC cells (5 × 10^4^ cells suspended in 2 μL PBS) into the subretinal space.

#### 2.9.14 *In vivo* tumor inhibition analysis

Thirty mice from the orthotopic UM model were randomly divided into six treatment groups (n = 5 per group): (1) Untreated; (2) Free PD-L1 Ab; (3) HANP/VP; (4) HANP/VP & PD-L1 Ab; (5) HANP/VP + Laser; and (6) HANP/VP + Laser & PD-L1 Ab. Treatments were administered on days 7 and 9. Mice received an equivalent VP dose of 5 mg/kg and PD-L1 Ab dose of 10 mg/kg. For the HANP/VP + Laser and HANP/VP + Laser & PD-L1 Ab groups, a 690-nm laser at 200 mW/cm^2^ was applied for 10 min. Eyeball diameters were measured using calipers, and tumor growth was monitored weekly using an In Vivo Imaging System (IVIS) after intraperitoneal injection of D-luciferin (15 mg/mL, 200 μL). On day 28 post-tumor cell inoculation, retinal function was analyzed in untreated and HANP/VP + Laser groups of mice using F-ERG (RetiMINER IV, IRC, China). Normal mice without tumors also served as a control.

On day 28 post–tumor inoculation, some mice in each group were euthanized. Eyeballs were collected for gross imaging and H&E staining to assess tumor growth, retinal structure, and overall ocular morphology. The eyeball volume was measured and calculated based on the length (the longest dimension) and width (the perpendicular dimension in the same plane) of the excised mouse eyeball using the formula: eyeball volume = 0.52 × length × width^2^. In addition, hearts, livers, spleens, lungs, and kidneys were collected from treated mice and stained with H&E to evaluate the systemic safety of HANP/VP. The remaining mice were monitored for BW and survival. If mice exhibited eyeball rupture, severe corneal ulceration, or inability to eat or drink, they were euthanized in accordance with humane endpoints. Monitoring continued until day 80, at which time BW and survival curves were plotted. Tumor tissue sections were subjected to Ki-67 and Caspase-3 IF staining to evaluate tumor cell proliferation and apoptosis. CRT staining was performed to assess ICD, and p-STING IF staining was used to evaluate activation of the cGAS–STING pathway.

#### 2.9.15 *In vivo* immune activation

Thirty mice from the orthotopic UM model were randomly divided into six treatment groups (n = 5 per group): (1) Untreated; (2) Free PD-L1 Ab; (3) HANP/VP; (4) HANP/VP & PD-L1 Ab; (5) HANP/VP + Laser; and (6) HANP/VP + Laser & PD-L1 Ab. Treatments were administered on days 7 and 9. Mice received an equivalent VP dose of 5 mg/kg and PD-L1 Ab dose of 10 mg/kg. For the HANP/VP + Laser and HANP/VP + Laser & PD-L1 Ab groups, a 690-nm laser at 200 mW/cm^2^ was applied for 10 min. PD-L1 Ab was administered via intraperitoneal injection, and the others were by intravenous injection.

Mice in each group were euthanized on day 10 post-treatment. Tumor tissues and spleens were collected, dissociated into single-cell suspensions, and stained for 1 h at 4 °C with the appropriate fluorescence-labeled Abs (BioLegend CNS, Inc., San Diego, CA, USA). FCM was used to assess CD8^+^ T cells (CD45.2^+^CD3^+^CD8^+^) in the tumor and mature DCs (CD11c^+^CD80^+^CD86^+^) in the spleen. Tumor sections were IF stained for CD3, CD68, CD163, CD11b, CD11c, and CD83 to evaluate infiltration of T cells, M1 macrophages, M2 macrophages, myeloid cells, DCs, and mature DCs. Staining for CD31 was used to assess tumor vasculature, and staining for PD-L1 to evaluate PD-L1 protein expression within tumors. Mouse serum was collected, and IFN-γ, IFN-β1, and TNF-α levels were measured using enzyme-linked immunosorbent assay (ELISA) kits.

#### 2.9.16 Immune memory effect

A total of 5 × 10^4^ B16F10 cells were injected into the subretinal space of the right eye in mice to establish a primary tumor. Ten mice were randomly divided into two groups (n = 5 per group): (1) Untreated; and (2) HANP/VP + Laser & PD-L1 Ab (VP-equivalent dose of 5 mg/kg, PD-L1 Ab–equivalent dose of 10 mg/kg). Treatments were administered on days 7 and 9, with laser irradiation at 690 nm and 200 mW/cm^2^ for 10 min.

Five days after completion of treatment, 1 × 10^4^ B16F10 cells were injected into the subretinal space of the left eye in each group to establish a tumor rechallenge model [61,62]. On day 28, mice were euthanized. The proportion of CD8^+^ Tems (CD3^+^CD8^+^CD44^+^CD62L^low^) in the spleen was analyzed via FCM, and the left eyes were harvested for H&E staining to evaluate tumor growth.

#### 2.9.17 Statistical analysis

Two-group comparisons were performed using Student's *t*-test, multiple-group comparisons using one-way analysis of variance (ANOVA), tumor diameter growth using two-way ANOVA, and survival data using the log-rank test. Results are presented as mean ± standard deviation. (SD). *p* < 0.05 indicates statistical significance.

## CRediT authorship contribution statement

**Shuyang Zhang:** Data curation, Writing – original draft. **Meijiao Song:** Data curation, Formal analysis. **Jialu Zhang:** Data curation, Methodology. **Jianshu Bai:** Data curation. **Xinyu Cao:** Data curation. **Lei Zhu:** Conceptualization, Writing – review & editing. **Rui Tian:** Funding acquisition, Resources, Supervision, Validation, Writing – review & editing.

## Declaration of competing interest

The authors declare that they have no known competing financial interests or personal relationships that could have appeared to influence the work reported in this paper.

## Data Availability

Data will be made available on request.
