# Supplementary material for: Combination of YAP inhibition and photodynamic therapy induces dual DNA damage and activates STING pathway to enhance immunotherapy in uveal melanoma
Source: Redox Biol. 2025 Dec 6;89:103965. doi: 10.1016/j.redox.2025.103965 (PMC12754387; doi:10.1016/j.redox.2025.103965)
Supplement: Multimedia component 1 [file mmc1.docx]

**Supplementary Information**

**Combination of YAP Inhibition and Photodynamic Therapy Induces Dual DNA Damage and Activates STING Pathway to Enhance Immunotherapy in Uveal Melanoma**

Shuyang Zhang^1,#^, Meijiao Song^1,#^, Jialu Zhang^1^, Jianshu Bai^1^, Xinyu Cao^1^, Lei Zhu^2,*^, Rui Tian^1,*^

1. Department of Ophthalmology, the Second Hospital of Jilin University, Changchun, Jilin Province, 130000, China;
2. Winship Cancer Institute, Department of Surgery, Emory University School of Medicine, Atlanta, GA 30322, United States.

# These authors contributed equally to this manuscript.

* To whom correspondence should be addressed. E-mail: [tianrui@jlu.edu.cn](mailto:tianrui@jlu.edu.cn) and [lei.zhu@emory.edu](mailto:lei.zhu@emory.edu).

**Methods**

**VP Loading Efficiency and Loading Content Measurements**

The encapsulation efficiency (EE) and loading capacity (LC) were derived from the measured content of VP by HPLC (Waters, Milford, MA). Separation was achieved on a C18 column (4.6 × 250 mm, 5 μm) using a 30-min linear gradient of 10–65% acetonitrile/water mixture (containing 0.1% trifluoroacetic acid) at 1 mL/min. The VP content was calculated based on a predetermined standard curve (y = 10.16x + 0.01409, R² = 0.9986). The EE and LC were calculated as follows:

$$EE(\%)=\frac{Amount of VP in micelles}{Total amount of VP added}\times100\%$$

$$LC\left( \% \right)=\frac{Amount of VP in micelles}{Weight of micelles}\times100\%$$

**Characterization of HANP/VP**

Dissolve VP in DMSO, then dilute with ddH_2_O to a concentration of 0.01 mg/mL; the prepared HANP/VP solution contains VP at a concentration of 0.01 mg/mL. The absorbance spectra of VP and HANP/VP were measured and analysed comparatively using a Multiskan GO microplate reader (Thermo Fisher Scientific, MA, USA). The diameter and zeta potential of HANP/VP were determined at 25°C using a Malvern Zetasizer Nano ZS (Malvern Instruments, Malvern, UK). To assess drug stability, HANP/VP was dissolved in ddH₂O, PBS, and DMEM containing 10% fetal bovine serum (FBS). Samples were maintained at 25°C with particle size measured daily for seven consecutive days. Photographic observations of precipitation were conducted on days 1 and 7.


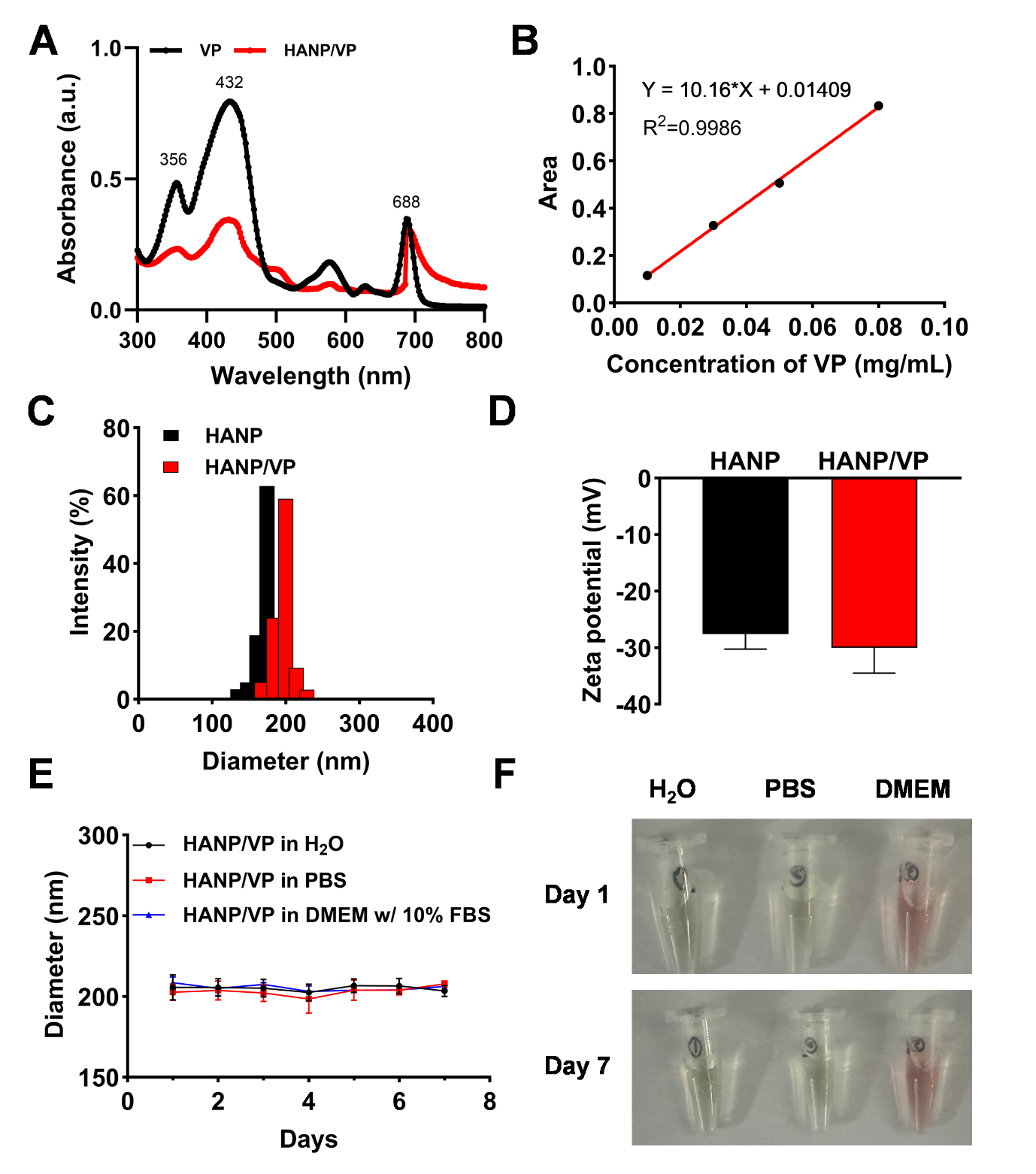


**Figure. S1. Characterization of HANP/VP. (A)** UV–vis–NIR spectrum of VP and HANP/VP. The characterized absorbances at 345, 435, and 682 nm of VP were detected in the HANP/VP complex. **(B)** The standard curve of VP as determined by HPLC. **(C)** The diameters of HANP and HANP/VP were determined by DLS. **(D)** Zeta potentials of HANP and HANP/VP were measured as −28 ± 3 mV and −30 ± 4 mV, respectively. **(E, F)** Stability of HANP/VP in different buffers, including H2O, PBS, and DMEM containing 10% FBS. Diameters of HANP/VP were not changed significantly during 1 week of incubation at room temperature without precipitation.

**
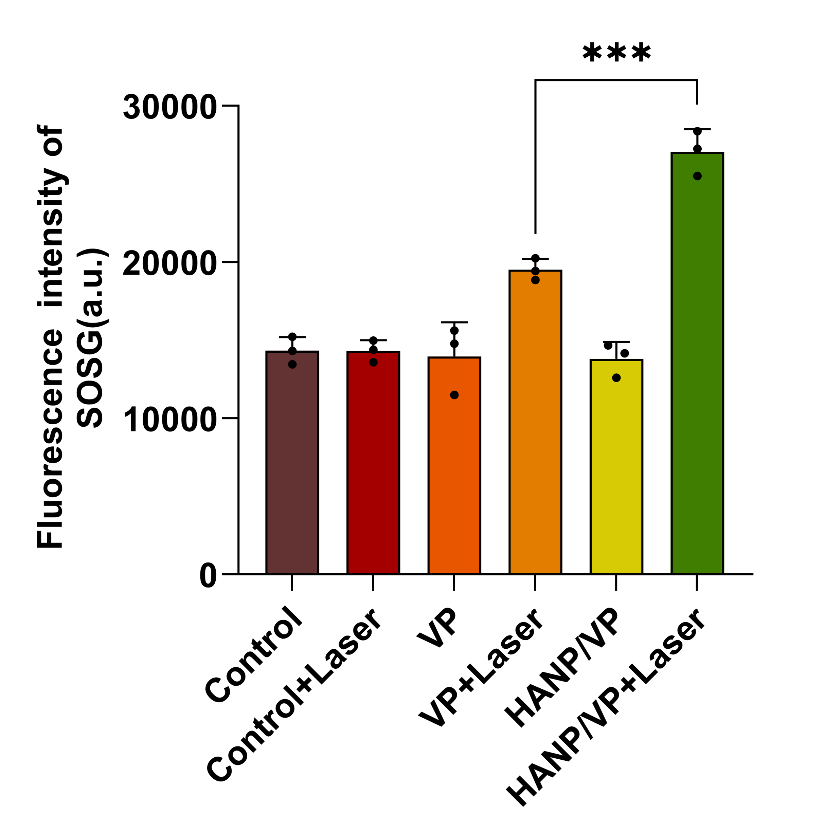
**

**Figure S2. The production of singlet oxygen (¹O₂) is measured by SOSG.** In the absence of ¹O₂, the anthracene part quenches the fluorescein's fluorescence through electron transfer, resulting in weak blue fluorescence. When SOSG encounters O₂, the anthracene moiety reacts with it, forming an endoperoxide, the fluorescence of which can be detected. The free VP did not generate a significantly higher amount of ¹O₂, even in the presence of laser irradiation, compared to the untreated control cells due to its poor biocompatibility and limited internalization intracellularly. HANP/VP produced a large amount of ¹O₂ after laser irradiation. n=3 wells/group. *** *p*<0.0001.

**
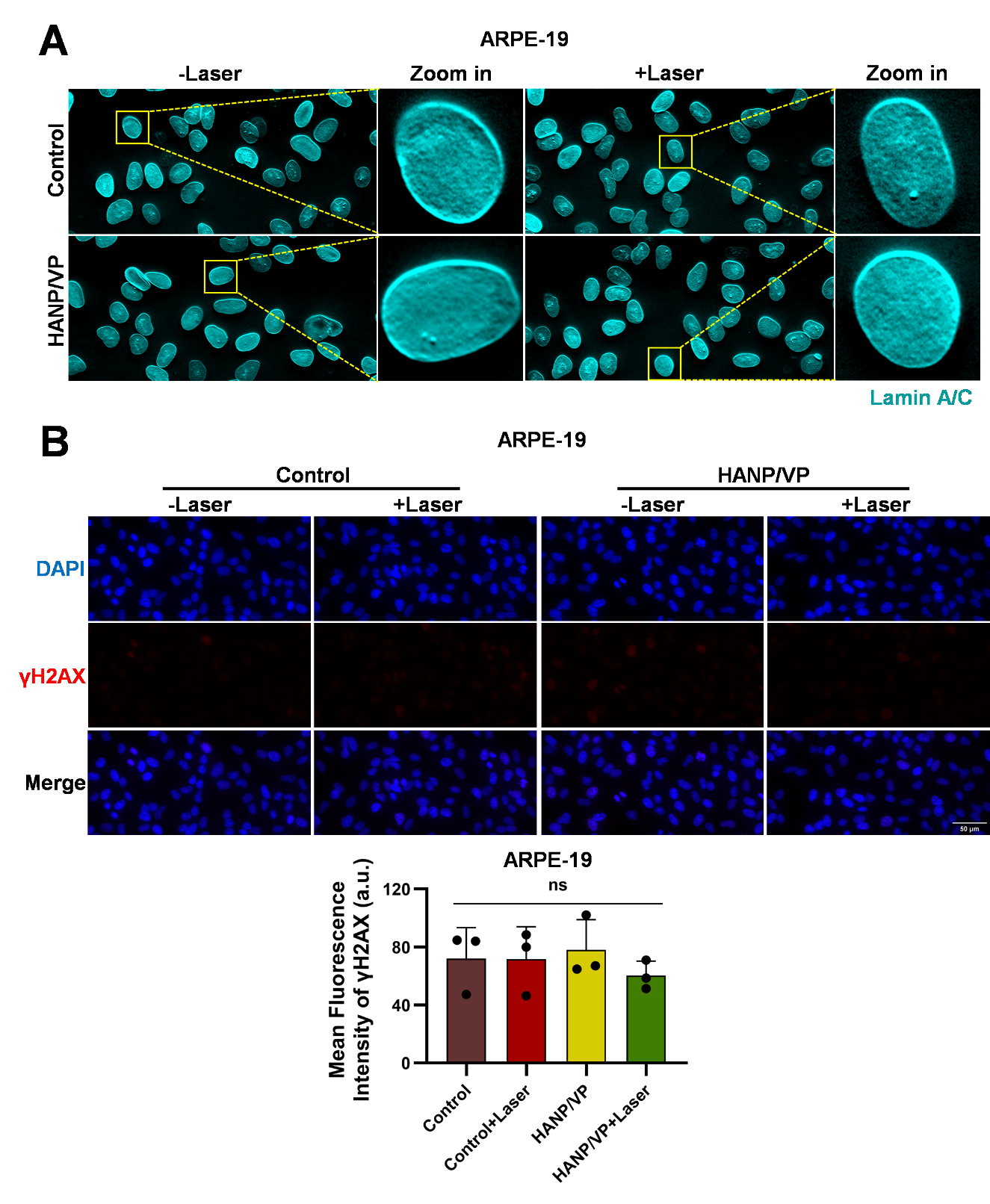
**

**Figure. S3. Effects of HANP/VP on the nucleus of ARPE-19 cells.** **ARPE-19 cells were treated with 1 μM HANP/VP for 12 h, followed by laser irradiation (690 nm, 100 mW/cm^2^, 5 min), with the non-laser irradiated group set up accordingly. (A)** Immunofluorescence staining of Lamin A/C to observe nuclear morphology in ARPE-19 cells. No nuclear deformation was observed in all groups (n=3). (**B)** γH2AX immunofluorescence staining to evaluate nDNA damage levels in ARPE-19 cells. No nDNA damage signal was observed in all groups (n=3). Statistical significance in **(B)** was determined by one-way ANOVA (^ns^*p* > 0.05).

**
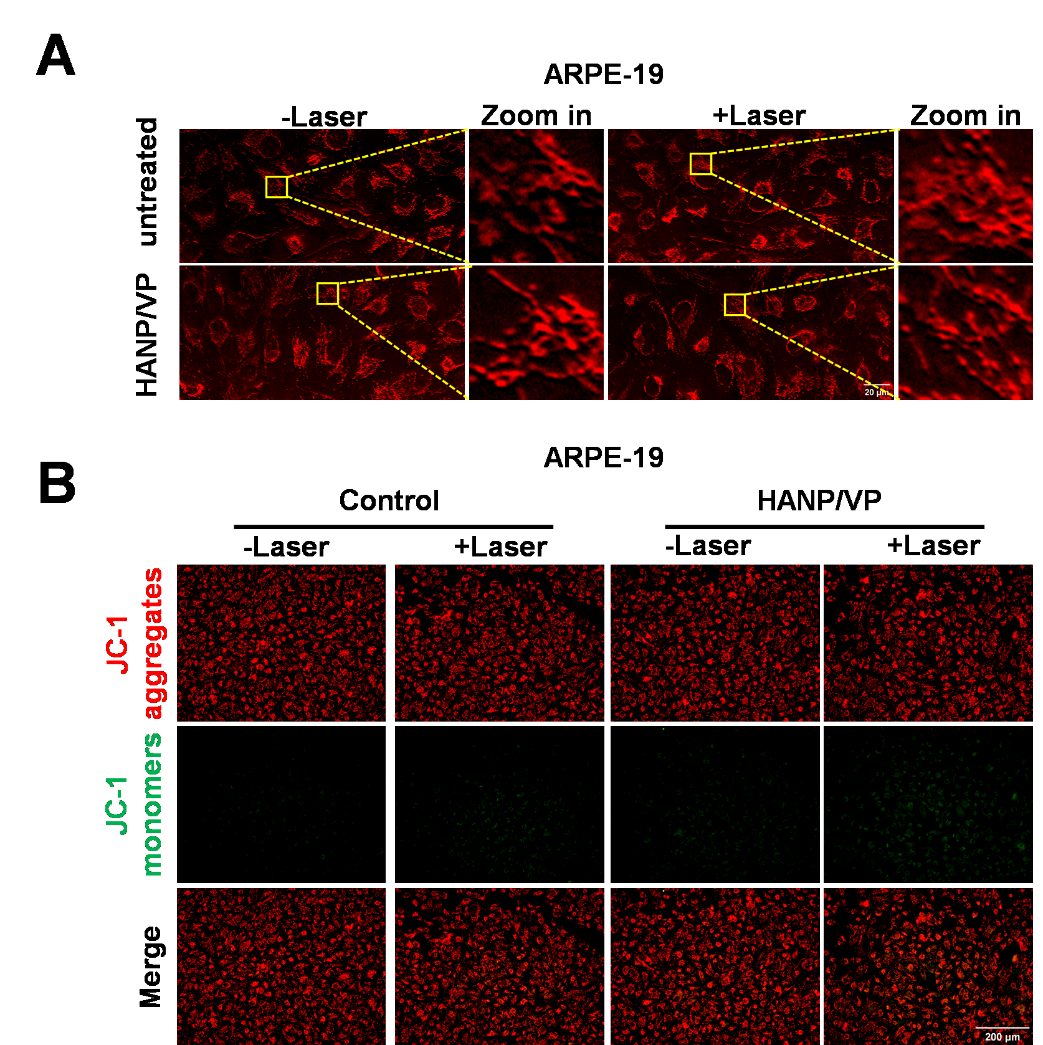
**

**Figure. S4. Effects of HANP/VP on mitochondria in ARPE-19 cells. ARPE-19 cells were treated with 1 μM HANP/VP for 12 h, followed by laser irradiation (690 nm, 100 mW/cm^2^, 5 min), with the non-laser irradiated group set up accordingly. (A)** Mitotracker staining to observe mitochondrial morphology in ARPE-19 cells. No disruption of mitochondrial morphology was observed in all groups (n=3). (**B)** JC-1 staining to evaluate changes in mitochondrial membrane potential in ARPE-19 cells. JC-1 was present as aggregates (red) in all groups, indicating normal mitochondrial function (n = 3).

**
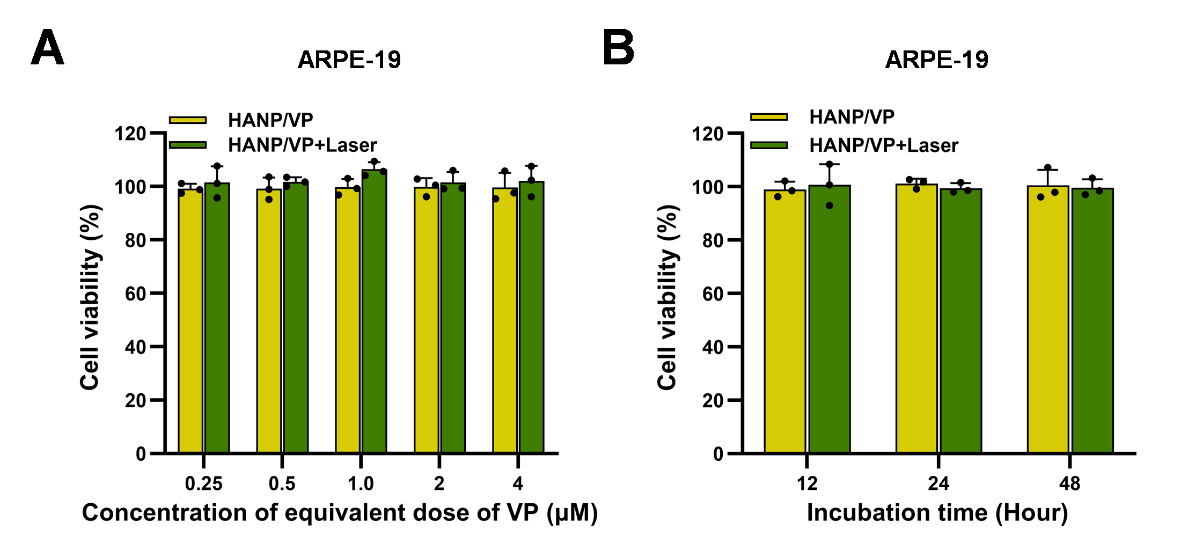
**

**Figure. S5. Cytotoxicity of HANP/VP toward ARPE-19 cells.** (**A)** ARPE-19 cells were treated with VP-equivalent concentrations of 0.25, 0.5, 1, 2, and 4 μM HANP/VP for 12 h followed by laser irradiation (690 nm, 100 mW/cm^2^) for 5 min, with a corresponding non-irradiated group. Cell viability was determined by CCK-8 assay (n=3). (**B)** ARPE-19 cells were treated with 1 μM HANP/VP for 12, 24, and 48 h, followed by laser irradiation (690 nm, 100 mW/cm2, 5 min), with the non-laser irradiated group set up accordingly. Cell viability was determined by CCK-8 assay (n=3).


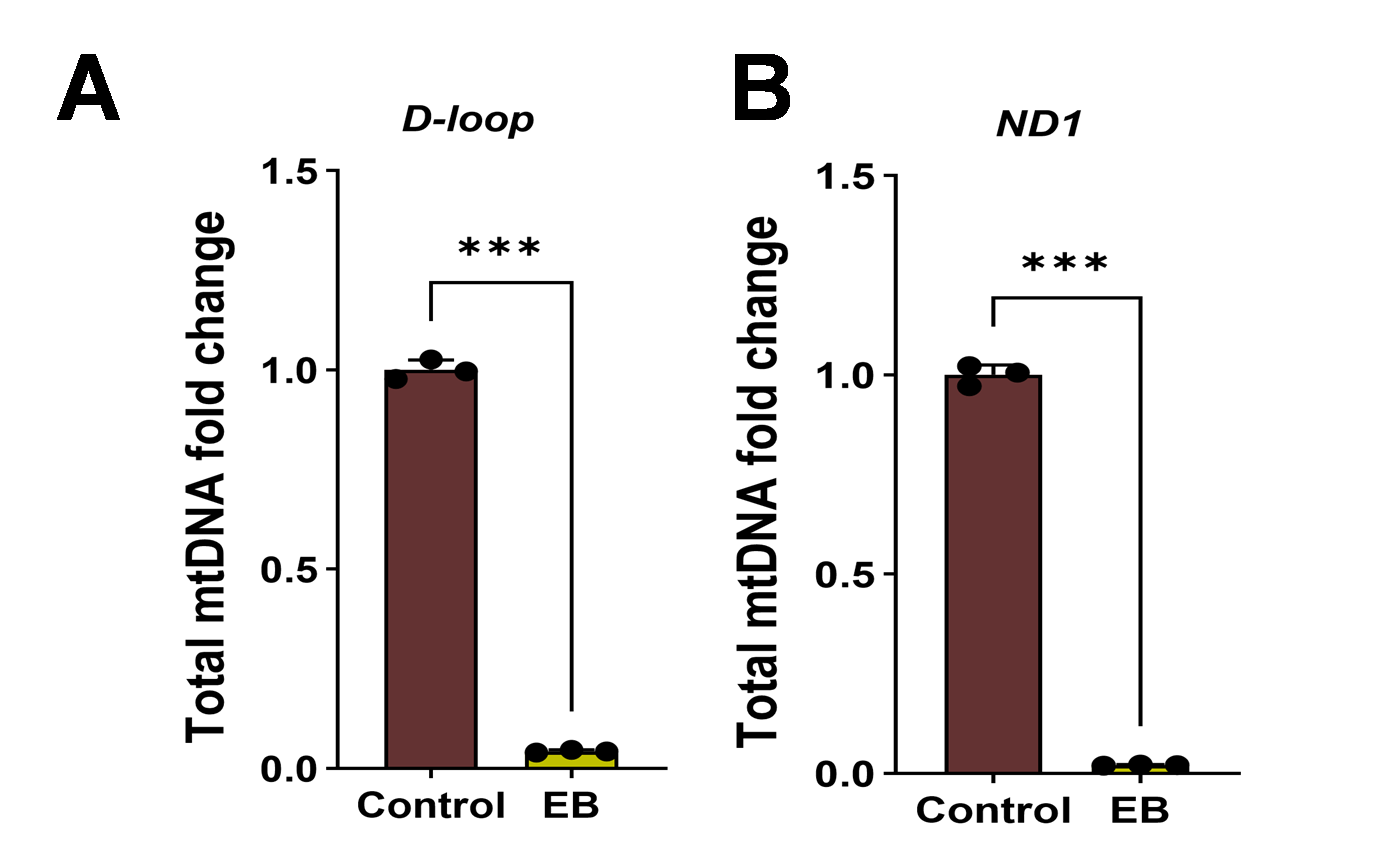


**Figure. S6. mtDNA replication and transcription are diminished by EB, which is a compound that intercalates with mtDNA, causing the depletion of both the mitochondrial D-loop (A) and ND1 (B) .**


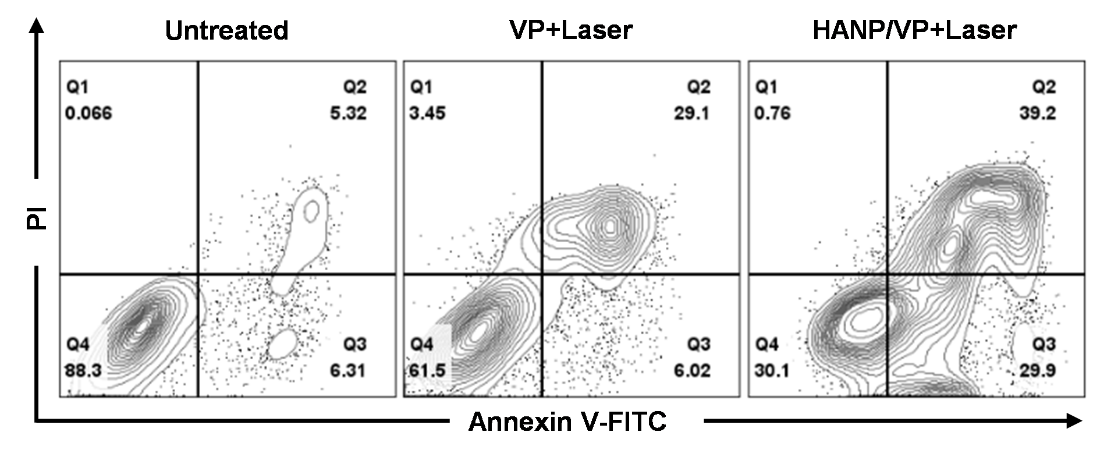


**Figure. S7. Representative flow cytometry results assessing apoptosis by Annxin V/PI staining are shown B16F10 cells were treated with VP (1 μM) or HANP/VP (VP equivalent dose of 1 μM) for 12 h, then combined with laser irradiation (690 nm, 100 mW/cm^2^, 5 min).**

**
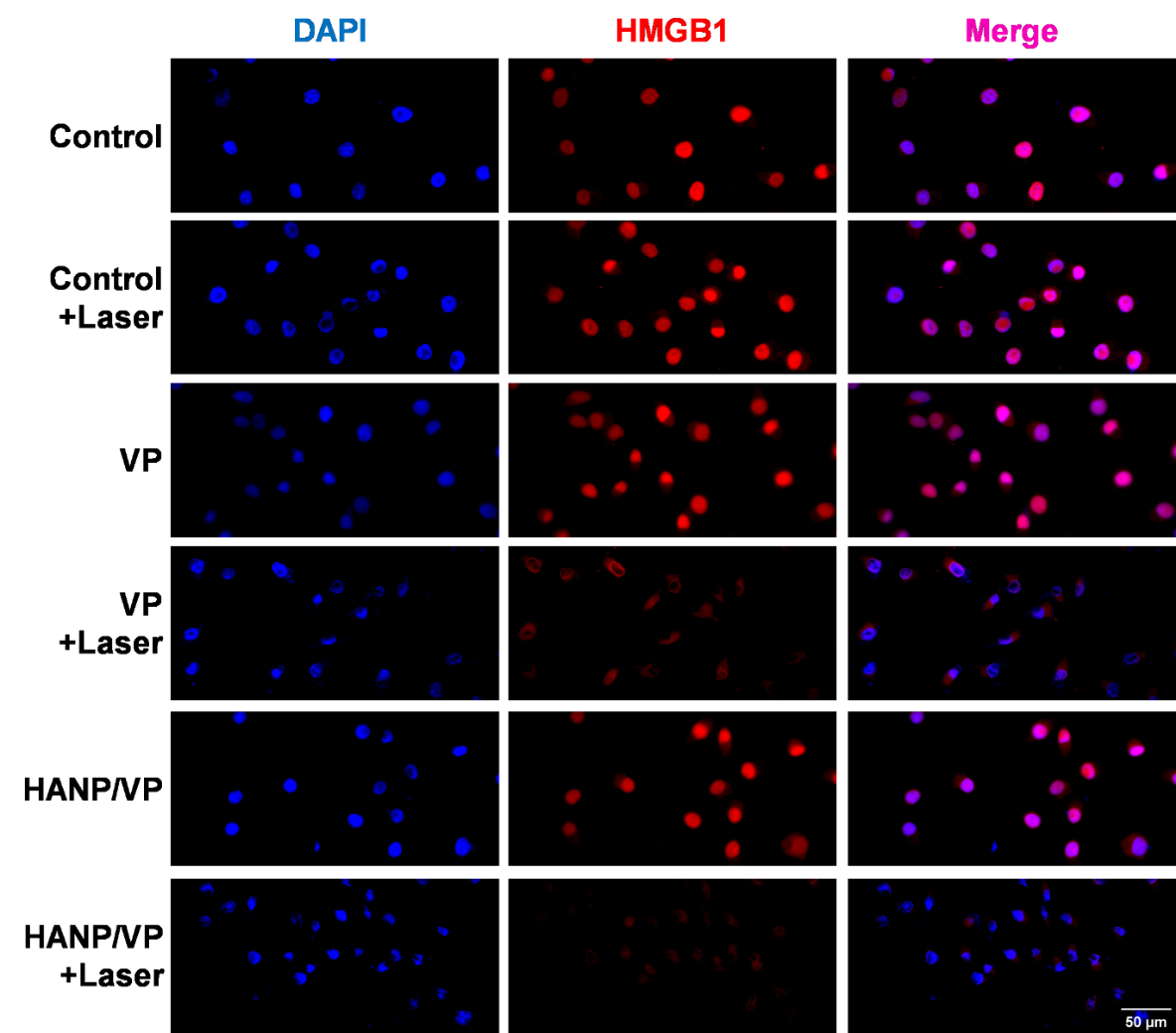
**

**Figure. S8. Immunofluorescence staining was performed to assess the expression of HMGB1 in B16F10 cells after corresponding treatments.**

**
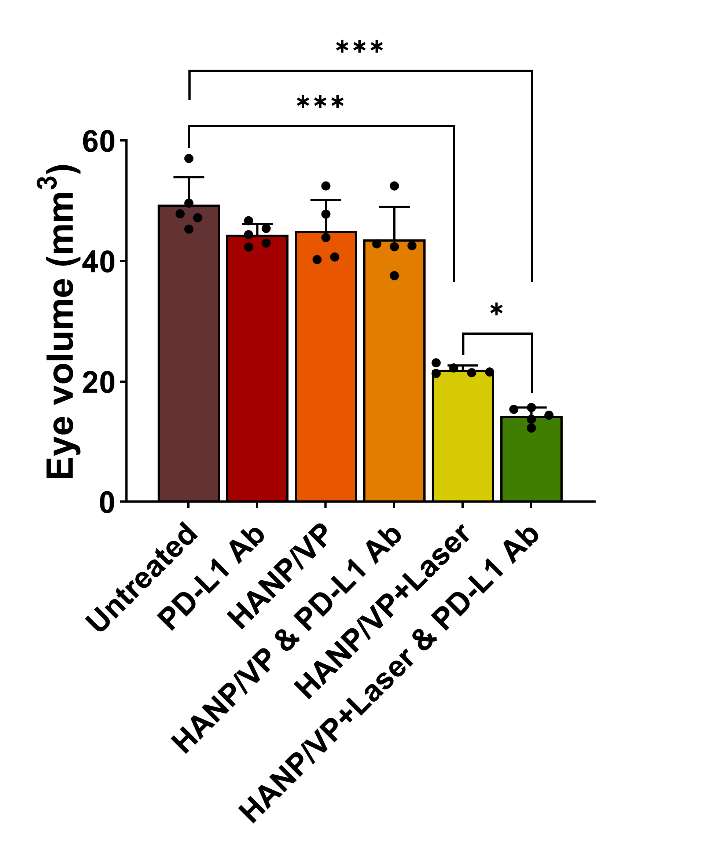
**

**Figure. S9. On day 28 after tumor cell inoculation, the volume of the burdened tumor eyeballs excised from mice in each group (n = 5 mice/group). Statistical significance was determined by one-way ANOVA, (*p< 0.05, **p < 0.01, ***p < 0.001)**

**
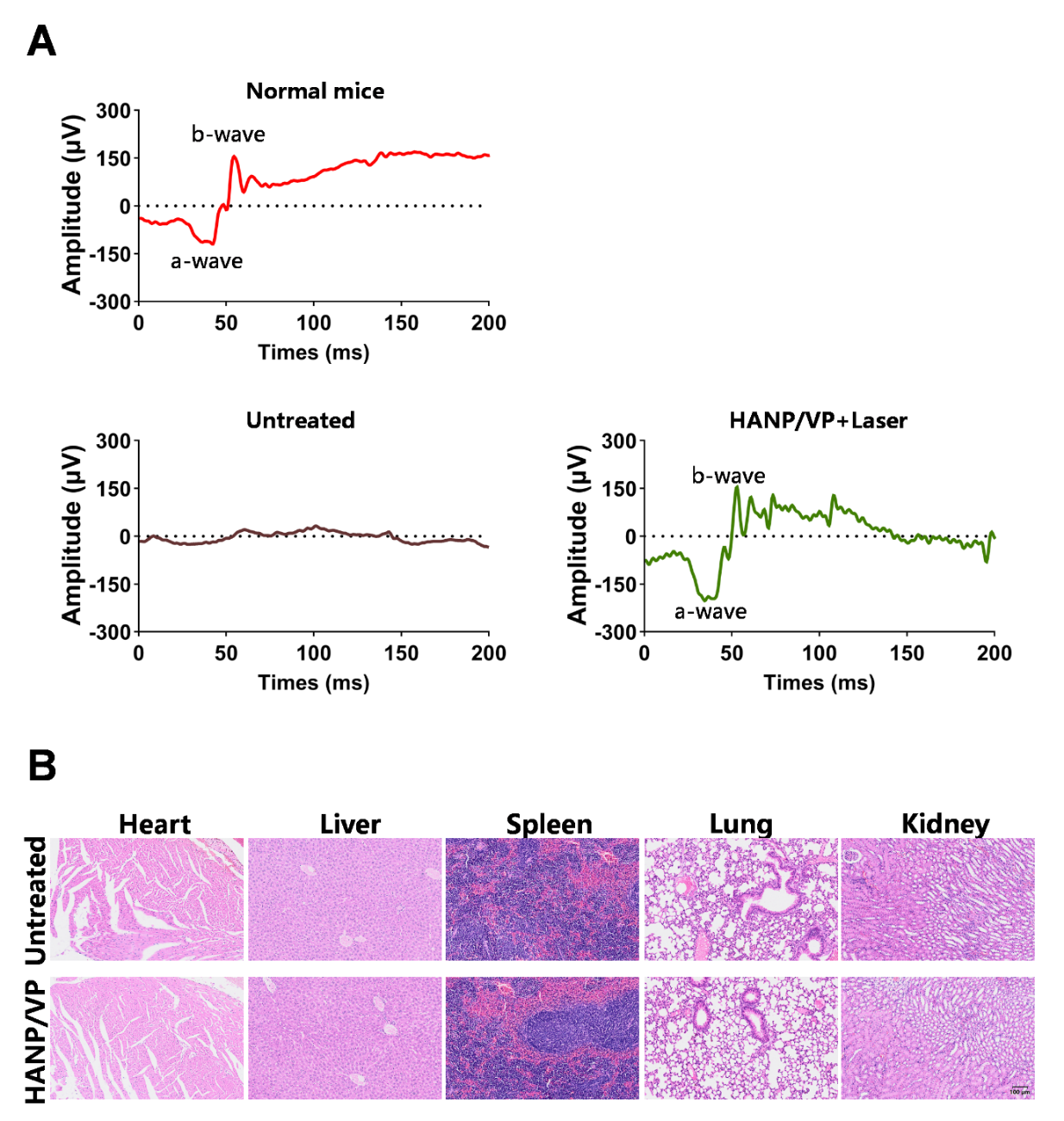
**

**Figure. S10. HANP/VP exhibits favorable ocular and systemic safety. (A)** Retinal function in each treatment group was assessed using F-ERG, where the first negative wave represents the a-wave (reflecting retinal photoreceptor cell activity) and the first positive wave represents the b-wave (reflecting retinal bipolar cell activity). The HANP/VP+Laser group demonstrated a-wave and b-wave characteristics similar to those observed in the Normal mice group, indicating preserved retinal function in these mice. **(B)** The HE staining of normal organs after HANP/VP treatments.


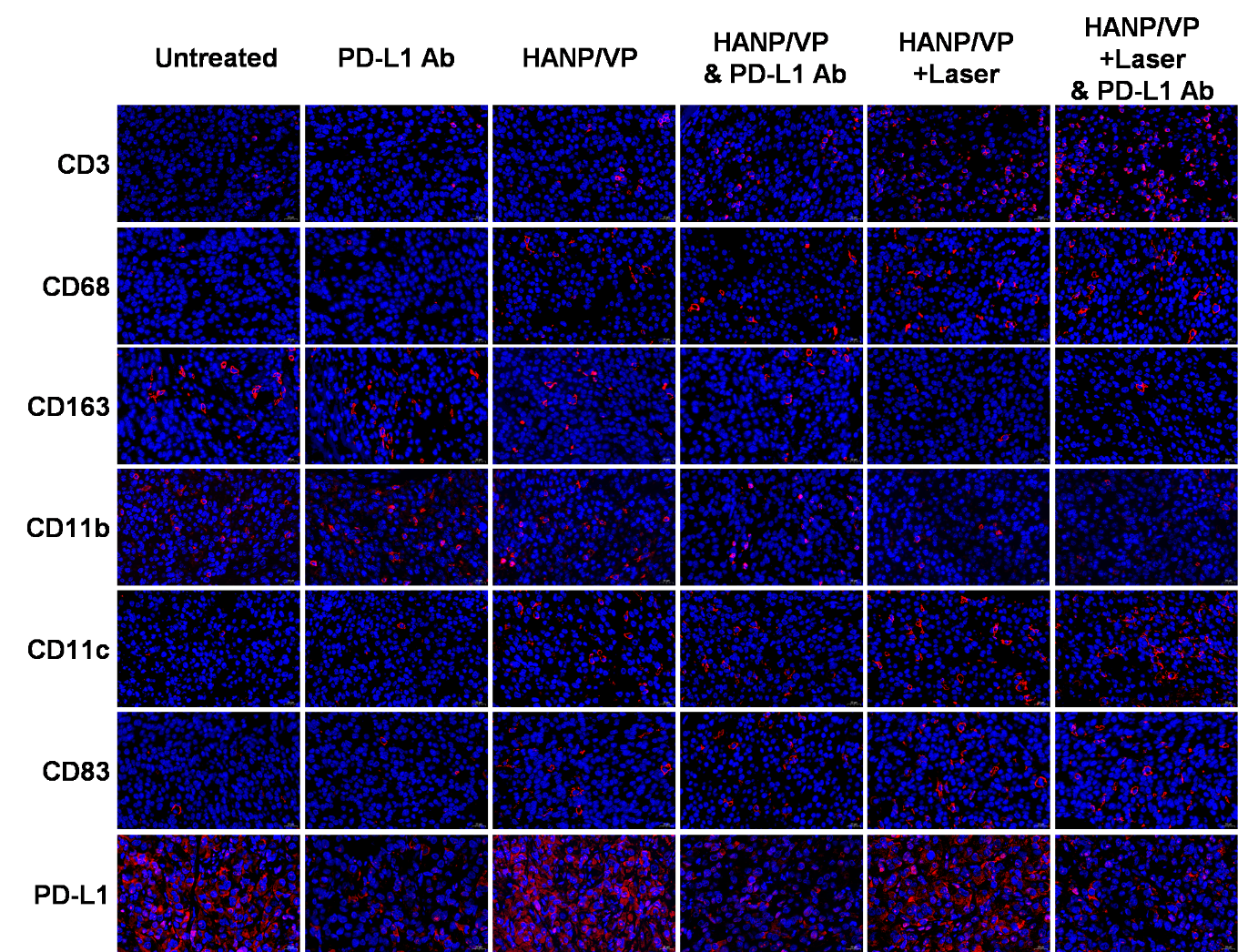


**Figure. S11.** **Immunofluorescence staining of immune active cells and immune suppressive cells, including CD3, CD68+ M1 macrophage, CD163+ M2 macrophage, CD11b+ myeloid-derived cells in tumor tissues, CD11c+ DCs, and CD83+ mature DCs.**
